# Supplementary material for: Predicting energy and stability of known and hypothetical crystals using graph neural network
Source: Patterns (N Y). 2021 Sep 30;2(11):100361. doi: 10.1016/j.patter.2021.100361 (PMC8600245; doi:10.1016/j.patter.2021.100361)
Supplement: Document S1. Supplemental experimental procedures and Figures S1–S13 [file mmc1.pdf]

Patterns, Volume *2*

Supplemental information

Predicting energy and stability of known and hypothetical crystals using graph neural network

Shubham Pandey, Jiaxing Qu, Vladan Stevanović, Peter St. John, and Prashun Gorai

Supplemental Experimental Procedures

1. Optimized Hyperparameters

The hyperparameters include parameters used to generate the crystal graphs, parameters of the neural networks, and parameters that control the training process. The hyperparameters are optimized through a train-validation process, on a fixed validation set. The following ranges of hyperparameters are searched: (1) batch size: 32–64, (2) embedding dimensions: 64–256, (3) number of message blocks: 4-8, and (4) learning rate: $1e^{-n}$, n = 3-5. The mean absolute error of total energy prediction is reduced by 0.005 eV/atom by using a weight decay compared to when not using it.

Table S1: List of optimized hyperparameters in this work

Hyperparameter	Optimized value
Batch size	64
Embedding dimension	256
Number of message blocks	6
Learning rate	$1e^{-3}$
Weight decay	$1e^{-5}$
Number of epochs	500

2. Performance of the Models Trained on Total Energy of ICSD Structures

To estimate the uncertainty in the mean absolute error (MAE) of total energy prediction, four different models are trained on the DFT total energy of ICSD structures. The uncertainty in the MAE is the standard deviation across the four models, each tested on a different hold-out test set.

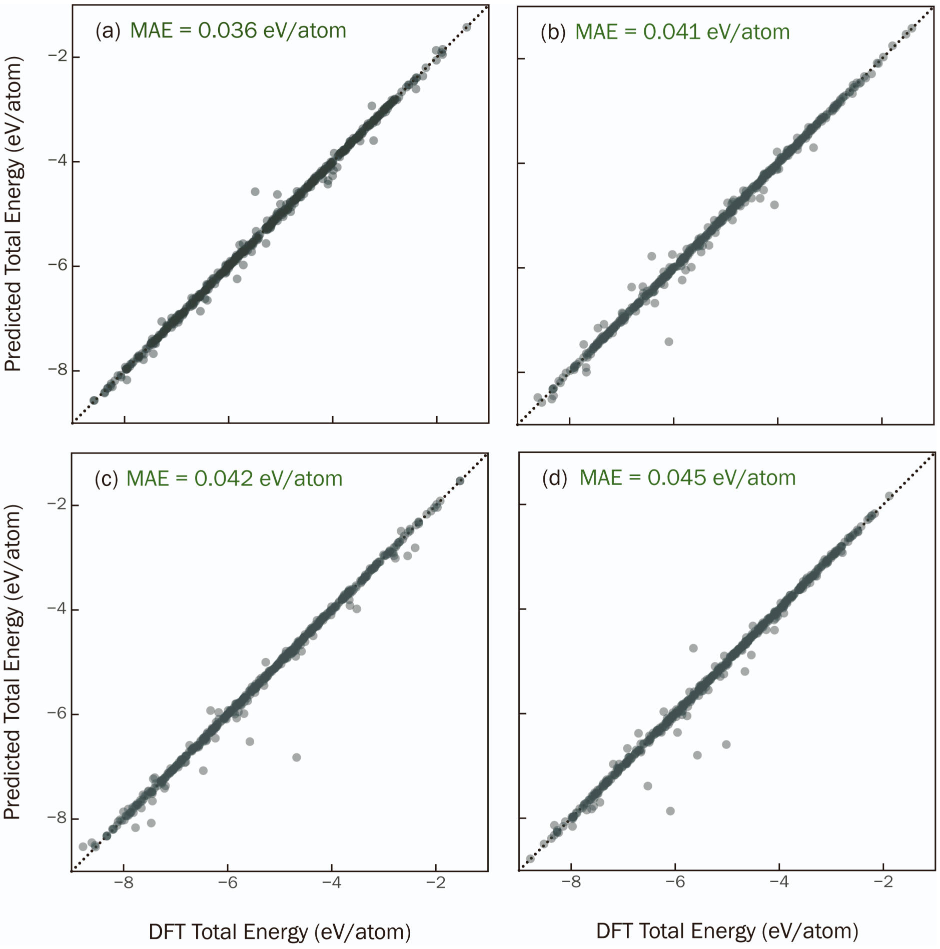

Figure S1: **Models trained on ICSD structures:** Convolutional neural networks trained on DFT total energy of ICSD structures from the NREL Materials Database. (a)-(d) Performance of the models trained and tested on four different sets of crystal structures. The mean absolute errors (MAEs) for the four different test sets are (a) 0.036 eV/atom, (b) 0.041 eV/atom, (c) 0.042 eV/atom, and (d) 0.045 eV/atom. The overall MAE across the four models is 0.041±0.005 eV/atom.

3. Performance of Model Trained on Total Energy of Hypothetical Structures

The model trained exclusively on the hypothetical structures is used to predict the total energy of the ICSD structures. Only a subset of ICSD structures, which contain the same 24 elements present in the hypothetical structure dataset, are chosen. The model poorly predicts the total energy of the ICSD structures with a large mean absolute error.

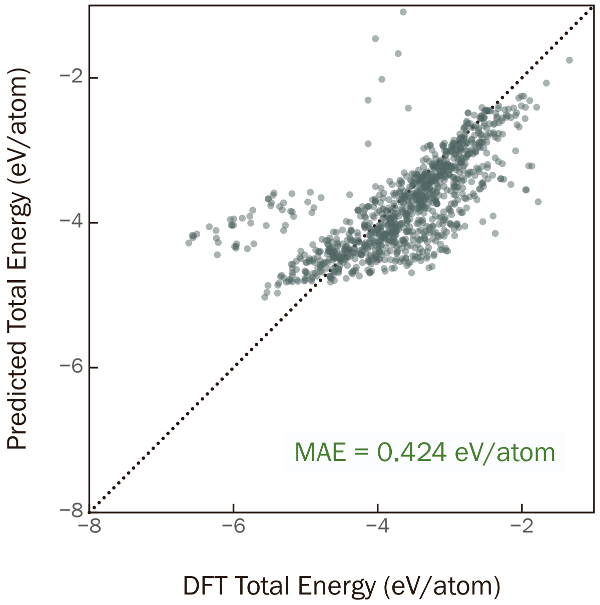

Figure S2: **Model trained on hypothetical structures:** The total energy of 1065 ICSD structures is predicted with the model trained on the hypothetical structures alone (see Section 2.2 in the main text). The predicted total energy has large errors compared to the DFT values. The mean absolute error (MAE) of the test set prediction is 0.424 eV/atom, suggesting the model is biased towards hypothetical structures.

4. Performance of the Hybrid Model Trained on Total Energy of ICSD and Hypothetical Structures

To estimate the uncertainty in the mean absolute error (MAE) of total energy prediction, four different models are trained on the DFT total energy of ICSD and hypothetical structures. The uncertainty in the MAE is the standard deviation across the four models, each tested on a different hold-out test set. The training, validation and test sets are chosen with no overlap of compositions for the hypothetical structures.

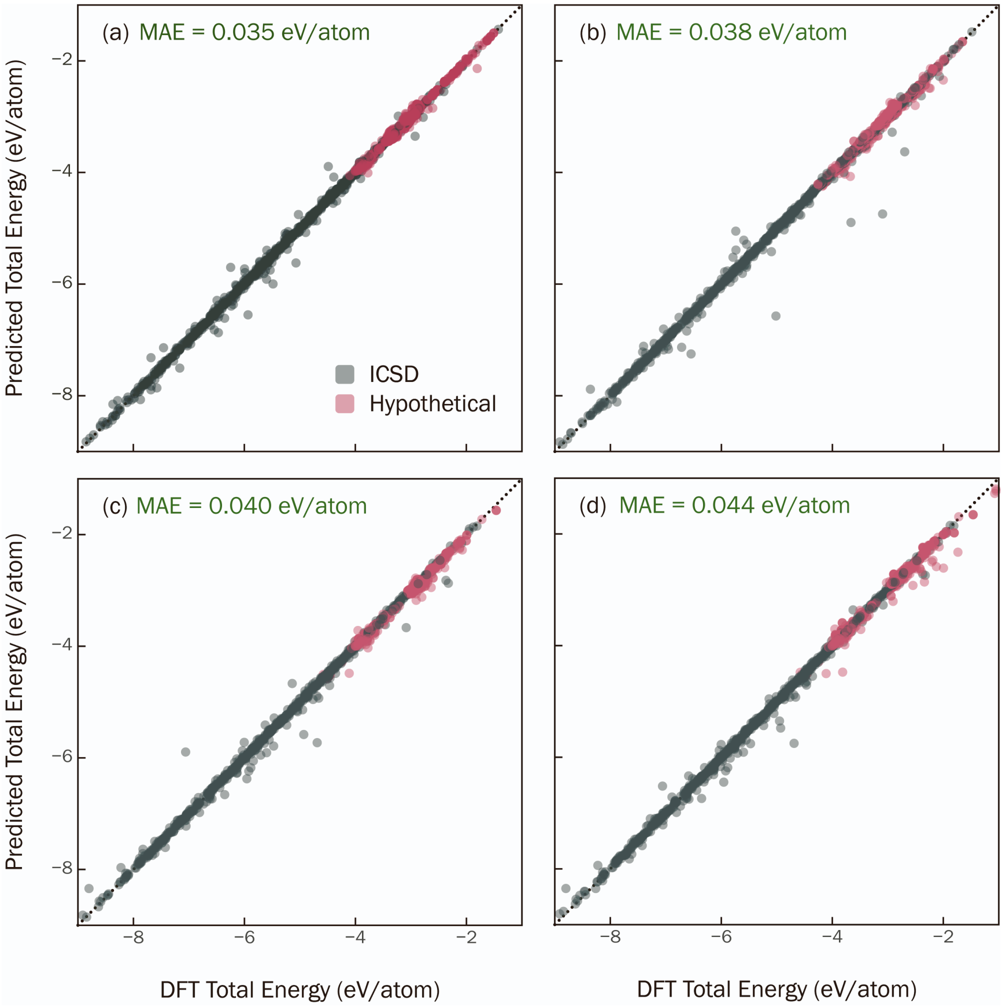

Figure S3: **Models trained on combined dataset:** Convolutional neural networks trained on DFT total energy of ICSD and hypothetical structures. (a)-(d) Performance of the models trained and tested on four different sets of crystal structures. The mean absolute errors (MAEs) for the four different test sets are (a) 0.035 eV/atom, (b) 0.038 eV/atom, (c) 0.040 eV/atom, and (d) 0.044 eV/atom. Gray(red) datapoints correspond to ICSD(hypothetical) structures The overall MAE across the four models is 0.040±0.005 eV/atom.

5. Learning Curve of the Model Trained on ICSD and Hypothetical Structures

A learning curve compares the performance of a model on a test set for varying number of training instances and therefore, can provide insights into whether a model is overfitted. The learning curve for the model trained on the "combined" dataset of ICSD anf hypothetical structures shows that: (1) there is a systematic improvement in the model performance with the number of training crystal structures, and (2) the minimum number of training structures to achieve an MAE<0.05 eV/atom is $\sim 2 \times 10^4$.

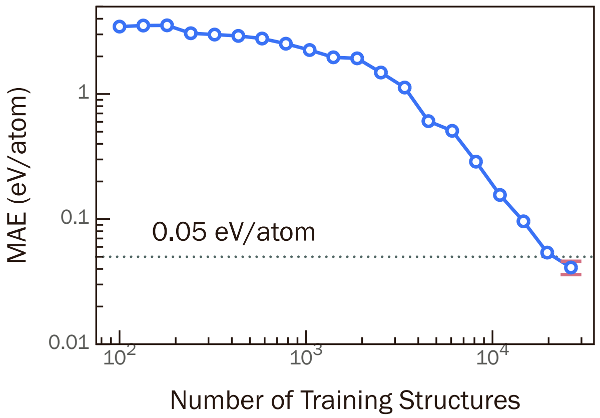

Figure S4: **Learning curve of model trained on combined dataset:** Learning curve for the hybrid model, showing that at least $2{\times}10^4$ crystal structures are required to achieve an MAE of <0.05 eV/atom.

6. Comparison of Prediction Accuracy

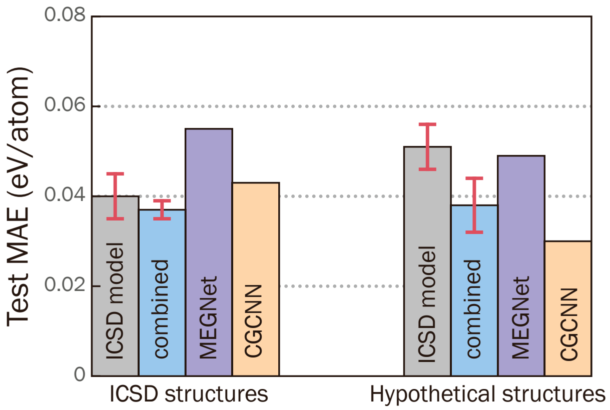

Figure S5: **Comparison of prediction accuracy:** Comparison of prediction MAE for ICSD and hypothetical structures of the model trained only on ICSD structures (Figure 1a in main text), model trained only on hypothetical structures (Figure 3a in main text), model trained on the combined dataset (blue), MEGNet model trained on the combined dataset (purple), and CGCNN model trained on the combined dataset (orange). For an equitable comparison, in all cases the same crystal structures are used in the training, validation, and test sets. The standard deviation (shown as error bars) is calculated from 4 different models with non-overlapping test sets.

7. Predicted Energy Rankings of MgO and ZnO Polymorphs

Figure 4 in the manuscript presents the energy rankings for different compositions in the hypothetical structures dataset. Here, we examine the energy rankings of two well-known binary compounds, MgO and ZnO, for which several experimentally realized and computationally proposed polymorphs are documented in the ICSD. There are 9 and 5 unique polymorphic structures reported for MgO and ZnO, respectively. The hybrid model correctly identifies the ground-state structures (rocksalt MgO, wurtzite ZnO) and also, satisfactorily ranks the other polymorphic structures.

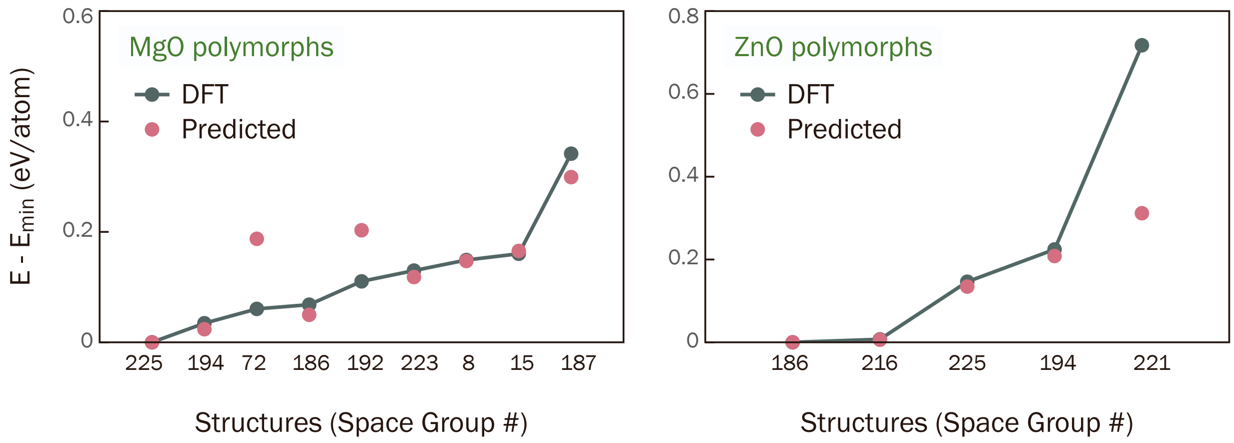

Figure S6: **Energy ranking of polymorphic structures:** Predicted relative energy ($E - E_{\mathrm{min}}$) of MgO and ZnO polymorphs reported in the ICSD is compared with DFT values. The model correctly identifies the known ground-state structures of both MgO (rocksalt, space group #225) and ZnO (wurtzite, space group #186).

8. Predicted Energy Ranking with GNN Models Trained on ICSD Structures

We train our GNN model, and MEGNet and CGCNN models on identical dataset consisting of only ICSD structures and compare the predicted energy rankings with DFT. The models consistently fail to rank structures in the correct order of their energies because of their bias towards the ground state structures. In particular, the models incorrectly label higher-energy structures as low energy.

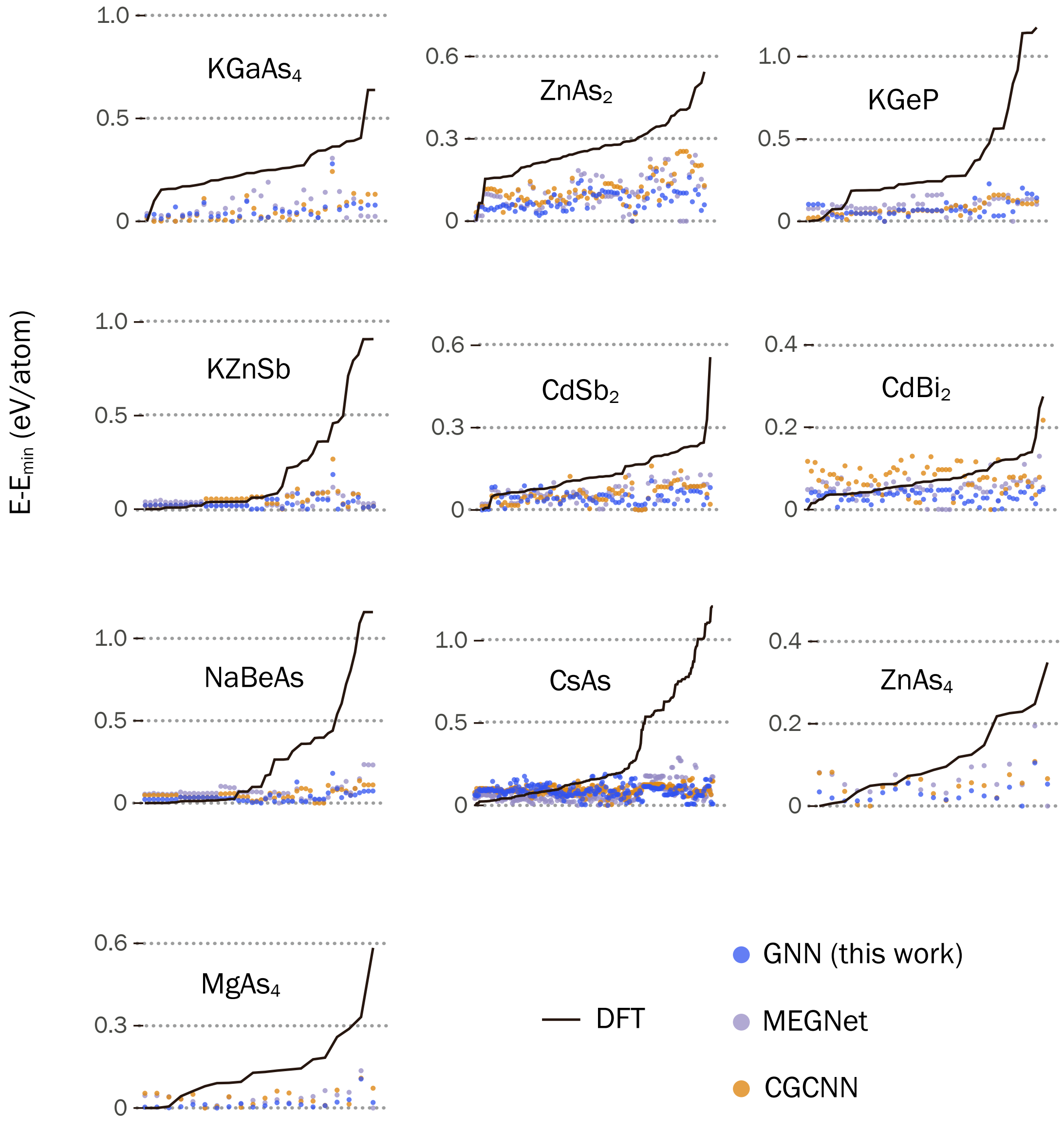

Figure S7: **Energy ranking of hypothetical structures:** Predicted relative energy ($E - E_{\min}$) of hypothetical structures of 10 different compositions from the test set in Figure 3(b) compared with DFT, using the models trained on ICSD structures. The x-axes represent different polymorphic structures of a given composition, which are generated through ionic substitution.

9. k-Nearest Neighbor Analysis of Ca_7Ge

The total energy of Ca_7Ge is severely underestimated (-0.545 eV/atom relative to the DFT value) by the hybrid model (Figure 3b). The intermetallic compound Ca_7Ge lies above the convex hull (see manuscript for details). To understand the source of the error, we perform a k-Nearest Neighbor (kNN) analysis of the elemental embeddings for all Ca and Ge sites in Ca_7Ge. From this analysis, we identify the first 10 NNs and their elemental identities. The Ca(*4b*) Wyckoff site has 9 NNs that are Ba atoms, while 1 NN is Sr. In contrast, the Ca(*24d*) Wyckoff site has 3 Ca NNs, 2 Sr, and 5 Ba. Moreover, the Ca-Ge bond lengths associated with the Ca(*4b*) site are larger compared to the Ca(*24d*) site. The Ge(*4a*) site has all 10 NNs that are Ge.

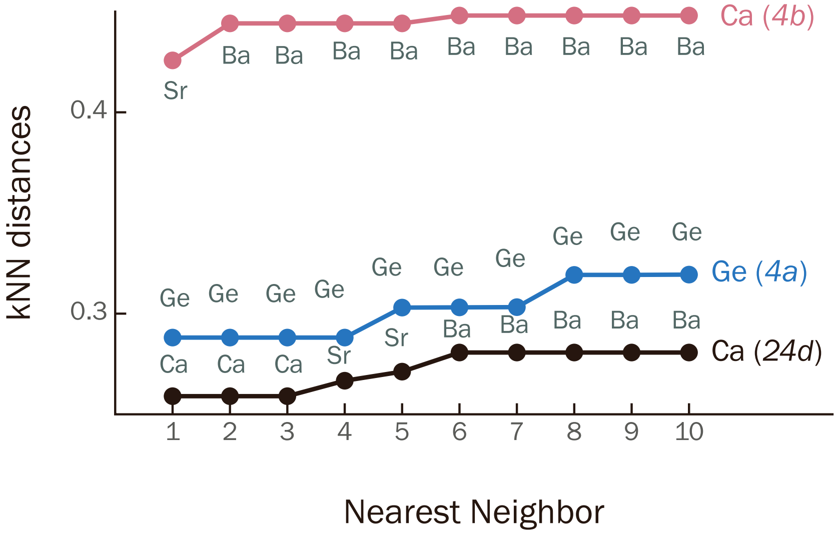

Figure S8: **Elemental nearest neighbor analysis of Ca_7Ge:** k-Nearest Neigbor (kNN) distances of the first 10 nearest neighbors for each Ca (*4b*, *24d* and Ge (*4a*) Wyckoff sites in Ca_7Ge. The elemental identities of the 10 nearest neighbors for each Wyckoff site are labelled.

10. Site Energies and t-SNE Projections: $Na_{17}Al_5O_{16}$, $Na_{14}Al_4O_{13}$

Chemical trends are identified by analyzing the probability density distribution of the elemental site energies (Figure 5) and t-SNE analyis of the elemental embeddings (Figure 6). In some cases, there can be a departure from the general trends. For example, some of the Na sites in $Na_{17}Al_5O_{16}$ (space group #8) and $Na_{14}Al_4O_{13}$ (space group #14) are 3-fold and 4-fold coordinated with elemental site energies in the "tail" of the oxides (near the peak of pnictides) energy distribution. The elemental embeddings for those same Na sites lie in the pnictogen cluster in the t-SNE projection. The other Na sites that lie closer to the peak of the oxides energy distribution (in the oxides cluster in t-SNE projection) are 5-fold coordinated.

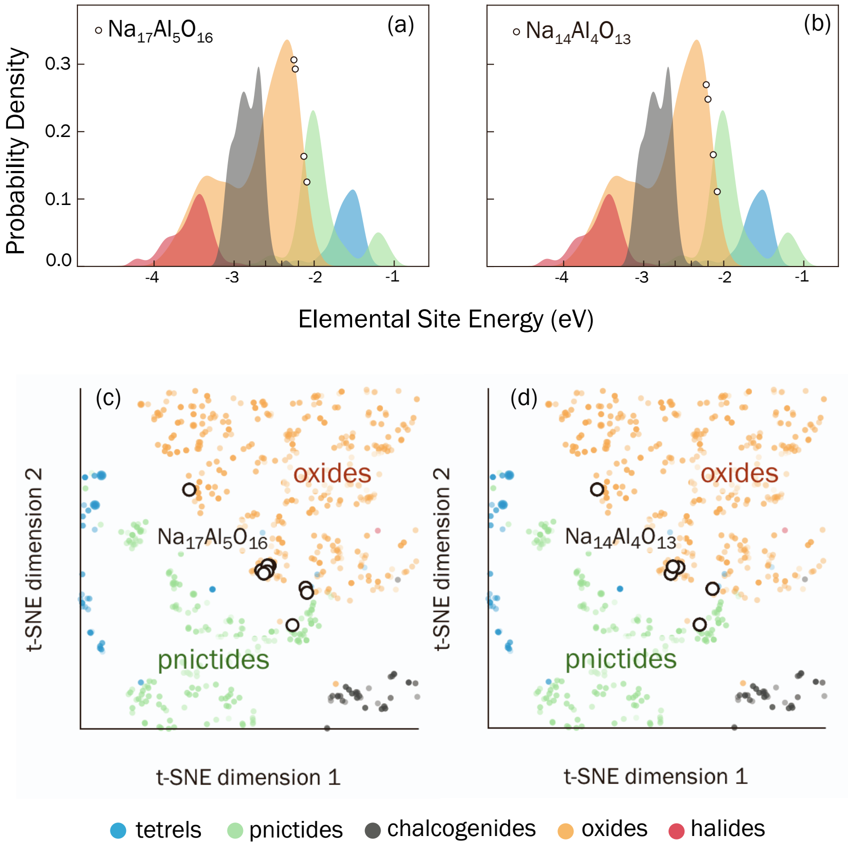

Figure S9: **Elemental site energies of $Na_{17}Al_5O_{16}$ and $Na_{14}Al_4O_{13}$:** Elemental site energy and t-SNE projection of elemental embedding of Na sites in (a, c) $Na_{17}Al_5O_{16}$ and (b, d) $Na_{14}Al_4O_{13}$ are marked with open circles.

11. Phase Stability Assessment with GNN Model

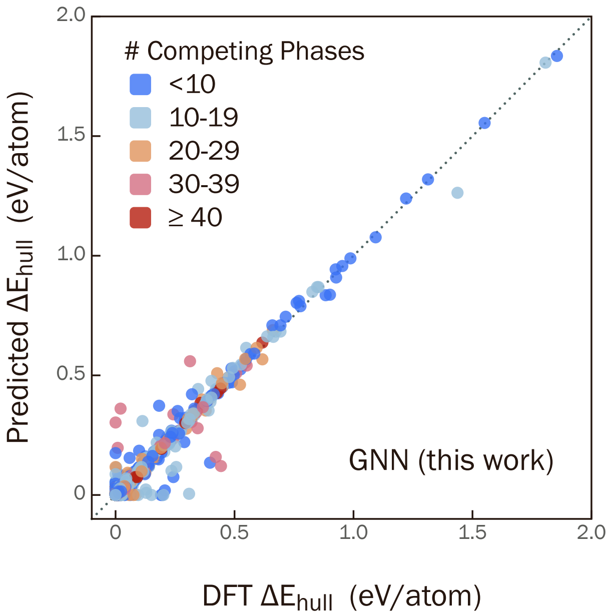

Figure S10: **Phase stability predicted with GNN:** Comparison of energy above the convex hull (ΔE_{hull}) predicted with our GNN model (trained on the combined dataset) and with DFT for 1794 ICSD compounds. The color scheme corresponds to the number of competing phases for each compound.

12. Phase Stability of Compounds Containing Group 5-10 Transition Metals

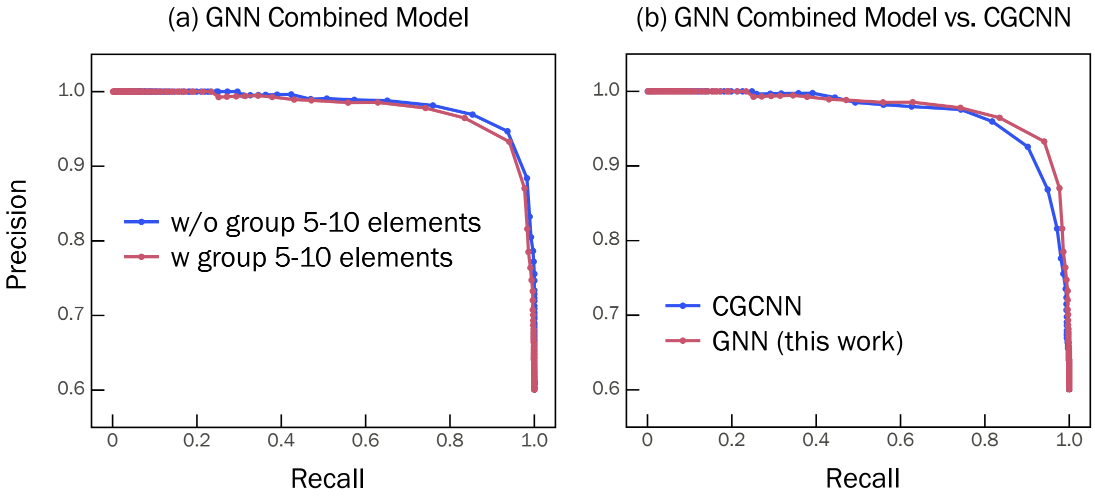

Figure S11: **Phase stability prediction of compounds with and without redox-active transition elements:** (a) PRC for phase stability predictions with our combined model compared for 761 compounds containing redox-active transition elements (groups 5-10) and 1033 compounds not containing groups 5-10 elements. (d) PRC for phase stability predictions of 1794 ICSD compounds (Figure S10) compared for our GNN model (trained on the combined dataset) and the CGCNN model (also, trained on the combined dataset). The performance of the re-trained CGCNN model is similar to our GNN model.

13. Comparison of ICSD and Combined Models in Predicting Phase Stability of Hypothetical Structures

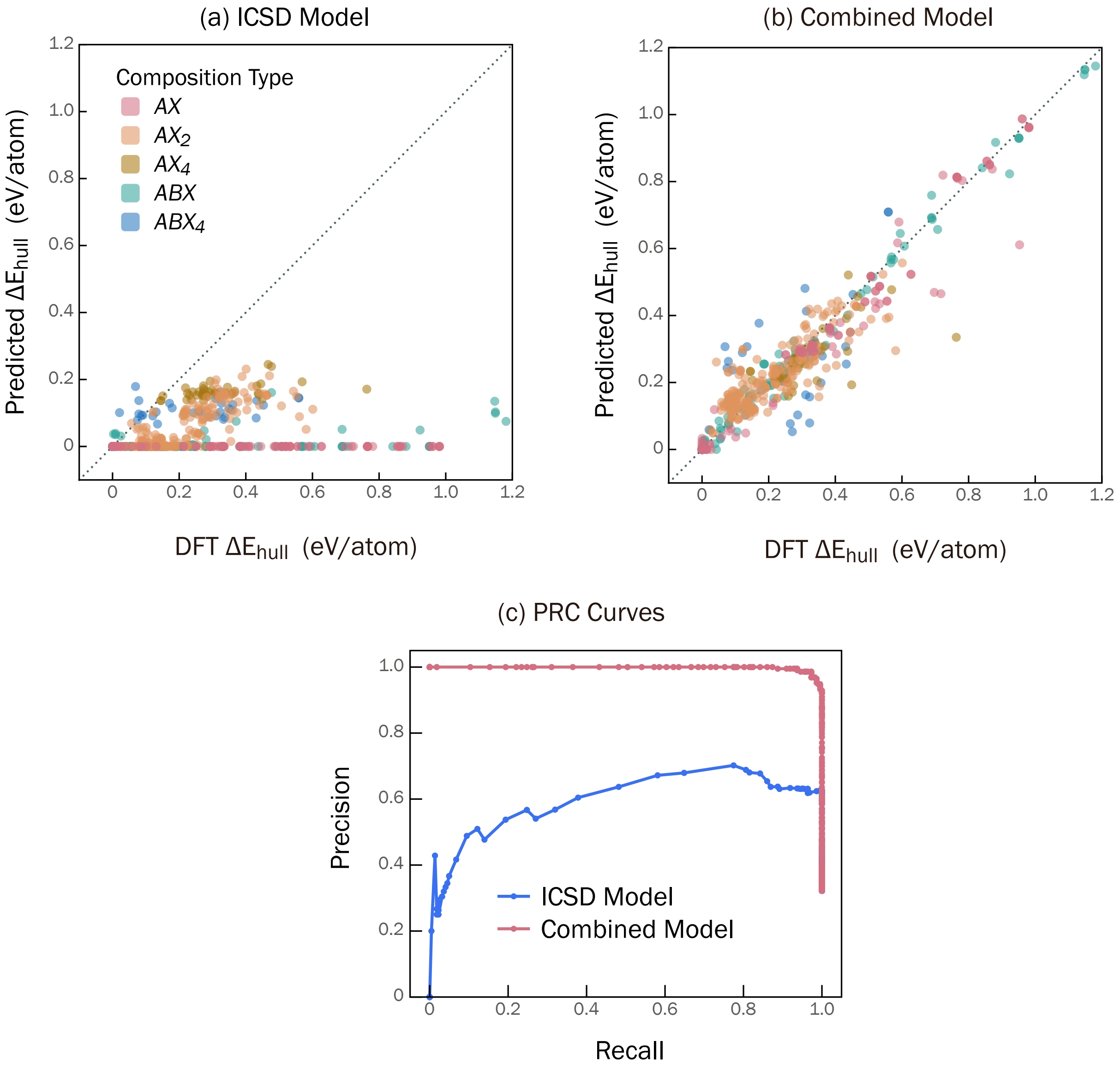

Figure S12: **Comparison of ICSD and combined models in predicting phase stability:** Phase stability of the hypothetical structures in Figure 4 of the main text. Predicted energy above the convex hull (ΔE_{hull}) compared with the corresponding DFT values for the (a) ICSD model, and (b) combined model. (c) Precision-Recall curves for the ICSD model and the combined model. Since the ICSD model is biased towards ground-state structures, it predicts significantly more false positives than the combined model. The area under the PRC curves (AU-PRC) for the ICSD and combined models are 0.60 and 0.99, respectively.

14. Comparison of GNN and Composition-only Models for Phase Stability

A precision-recall curve (PRC) provides a quantitative measure of the model's accuracy to classify a material as stable or unstable. The PRC for our GNN model prediction of phase stability of the 1033 compounds is compared with the PRC for stability predictions with the composition-based Magpie model. The area under the PRC (AU-PRC) is 1 for perfect classification and 0 for random guess. The AU-PRC for our GNN model is 0.98, which is significantly higher than that for Magpie model (0.78).

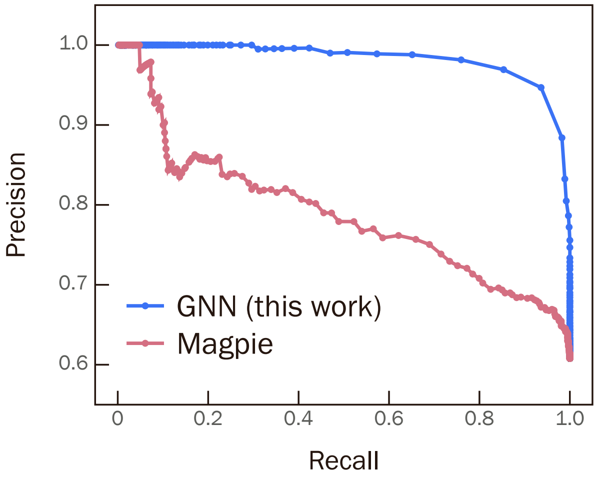

Figure S13: **Comparison of phase stability prediction with GNN and composition-only models:** Comparison of precision-recall curves (PRC) for phase stability predictions of 1033 ICSD compounds with our GNN model (trained on combined dataset) and the composition-based Magpie model.
