# Supplementary material for: Predicting energy and stability of known and hypothetical crystals using graph neural network
Source: Patterns (N Y). 2021 Sep 30;2(11):100361. doi: 10.1016/j.patter.2021.100361 (PMC8600245; doi:10.1016/j.patter.2021.100361)
Supplement: Document S2. Article plus supplemental information [file mmc2.pdf]

Article

Patterns

Predicting energy and stability of known and hypothetical crystals using graph neural network

Highlights

- A GNN is trained to predict total energy of ground-state and high-energy crystals
- The importance of a balanced training dataset is demonstrated
- The model ranks polymorphic structures of compounds with correct energy ordering

Authors

Shubham Pandey, Jiaxing Qu, Vladan Stevanović, Peter St. John, Prashun Gorai

Correspondence

peter.stjohn@nrel.gov (P.S.J.), pgorai@mines.edu (P.G.)

In brief

Discovery of new inorganic materials in unexplored chemical spaces necessitates calculating total energy quickly and with sufficient accuracy. Structure prediction and phase stability using *ab initio* methods is intractable to explore vast search spaces. We demonstrate the importance of a balanced training dataset of ground-state and higher-energy structures to accurately predict their total energies using a generic GNN architecture. We also demonstrate that the model satisfactorily ranks the structures in the correct order of their energies for a given composition.

Pandey et al., 2021, Patterns 2, 100361
November 12, 2021 © 2021 The Author(s).
https://doi.org/10.1016/j.patter.2021.100361

Patterns

CellPress
OPEN ACCESS

Article

Predicting energy and stability of known and hypothetical crystals using graph neural network

Shubham Pandey,[1] Jiaxing Qu,[2] Vladan Stevanović,[1] Peter St. John,[3,*] and Prashun Gorai[1,4,*]
[1]Department of Metallurgical and Materials Engineering, Colorado School of Mines, Golden, CO 80401, USA
[2]Mechanical Science and Engineering, University of Illinois, Urbana, IL 61801, USA
[3]National Renewable Energy Laboratory, Golden, CO 80401, USA
[4]Lead contact
*Correspondence: peter.stjohn@nrel.gov (P.S.J.), pgorai@mines.edu (P.G.)
https://doi.org/10.1016/j.patter.2021.100361

THE BIGGER PICTURE Large-scale *ab initio* calculations combined with advances in structure prediction have been instrumental in inorganic functional materials discovery. Currently, only a small fraction of the vast chemical space of inorganic materials has been discovered. The need for accelerated exploration of uncharted chemical spaces is shared by experimental and computational researchers. However, structure prediction and evaluation of phase stability using *ab initio* methods is intractable to explore vast search spaces. Here, we demonstrate the importance of a balanced training dataset of ground-state (GS) and higher-energy structures to accurately predict their total energies using a generic graph neural network. We demonstrate that the model satisfactorily ranks the structures in the correct order of their energies for a given composition. Together, these capabilities allow the model to be used for fast prediction of GS structures and phase stability and for the facilitation of new materials discovery.

1 2 3 4 5 **Proof-of-Concept:** Data science output has been formulated, implemented, and tested for one domain/problem

SUMMARY

The discovery of new inorganic materials in unexplored chemical spaces necessitates calculating total energy quickly and with sufficient accuracy. Machine learning models that provide such a capability for both ground-state (GS) and higher-energy structures would be instrumental in accelerated screening. Here, we demonstrate the importance of a balanced training dataset of GS and higher-energy structures to accurately predict total energies using a generic graph neural network architecture. Using ~16,500 density functional theory calculations from the National Renewable Energy Laboratory (NREL) Materials Database and ~11,000 calculations for hypothetical structures as our training database, we demonstrate that our model satisfactorily ranks the structures in the correct order of total energies for a given composition. Furthermore, we present a thorough error analysis to explain failure modes of the model, including both prediction outliers and occasional inconsistencies in the training data. By examining intermediate layers of the model, we analyze how the model represents learned structures and properties.

INTRODUCTION

With the advances in computing power and methodologies, computational chemistry and materials science have made great strides in accelerating discovery of molecules and materials with tailored properties.[1,2] The ability to perform large-scale *ab initio* calculations, in particular those based on density functional theory (DFT), has been instrumental in inorganic functional materials discovery.[3–7] However, computational searches have largely focused on *known* materials documented in crystallographic databases. Currently, there are ~200,000 entries in the Inorganic Crystal Structure Database (ICSD),[8] which represents only a small part (>10^{12} plausible compositions considering up to quaternary compounds)[9] of the vast chemical phase space of inorganic materials. The need for accelerated exploration of uncharted

This is an open access article under the CC BY license (http://creativecommons.org/licenses/by/4.0/).

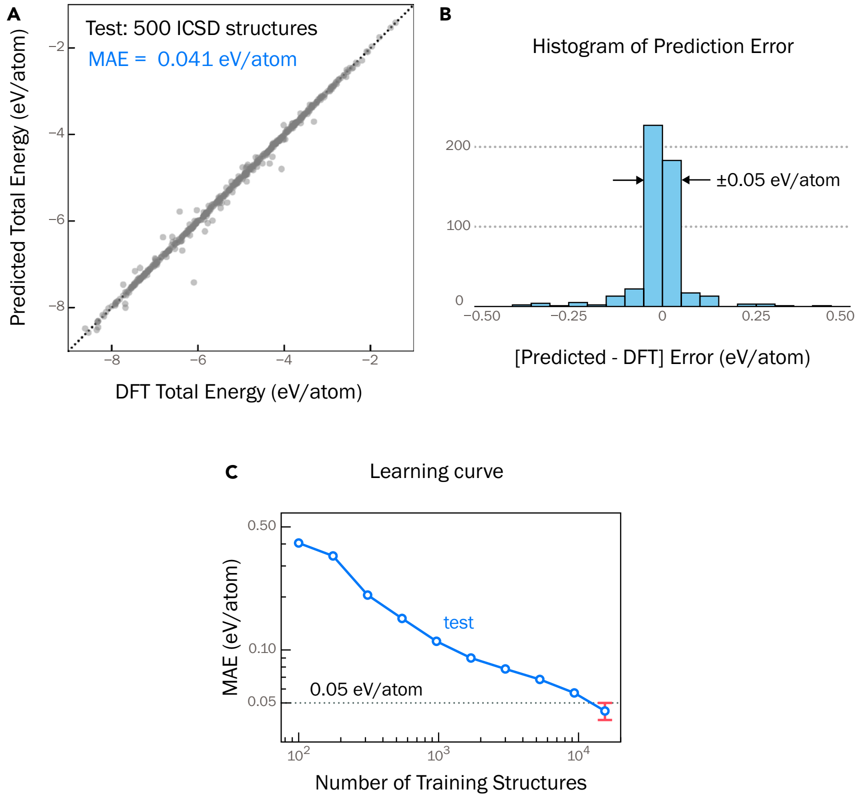

Figure 1. Model trained on ICSD structures
GNN model developed in this work trained on DFT total energy of ICSD structures from NREL Materials Database.[31]
(A) The model predicts DFT total energy of 500 held-out crystal structures with a MAE of 0.041 eV/atom (0.95 kcal/mol).
(B) Histogram of prediction errors (relative to DFT total energy) for the 500 test set structures; 82% of the structures are predicted within an error of ± 0.05 eV/atom.
(C) Learning curve shows that $>10^4$ training structures are needed to achieve MAE $\leq$0.05 eV/atom.

chemical spaces is shared by experimental and computational researchers.

The discovery of new inorganic compositions necessitates accurate structure prediction methods, which is a burgeoning field in itself. The general approach involves navigating the configuration space defined by the structural parameters, using a rapidly computable cost function such as total energy. The navigation of configuration space can use a variety of techniques, including simulated annealing,[10] genetic algorithms,[3,11] random structure searching,[12,13] structure prototyping,[14,15] and data mining.[16,17] In these techniques, total energy is often predicted with DFT, although force-field methods have also been used.[18,19] Thermodynamic phase stability, i.e. stability against decomposition, is another prerequisite in the search for new compositions. Formation enthalpy, calculated from DFT total energy, has proved immensely useful in assessing phase stability.[20–23] However, DFT total energy calculations are still computationally expensive to survey large chemical spaces with $>10^6$ compounds. Machine learning (ML) models have emerged as a surrogate for fast prediction of total energy, formation enthalpy, and phase stability.[24–26] Here, we develop a graph neural network (GNN) built upon existing architectures to predict the total energy of ground-state (GS) as well as hypothetical higher-energy structures generated for structure prediction.[16] In particular, we show that the effectiveness of any generic GNN to simultaneously predict the total energy of GS and higher-energy structures depends on the choice of training data. While most of the present literature on ML for predicting thermodynamic stability of materials is "model-centric" (i.e., focuses on improvements in model architecture), we show that the choice of training data is equally important.

Crystal graph convolutional neural networks (CGCNNs) have been developed to predict DFT total energy and formation enthalpy.[27–29] These deep learning models outperform traditional ML models with expert-designed feature representations. In a crystal graph, the atoms are represented by nodes and bonding interactions as edges connecting the nodes, which naturally takes into account the periodicity of crystal structures. Xie et al.[27] trained a CGCNN model on DFT-computed formation enthalpy of 46,744 crystal structures (predominantly from the ICSD) available in the Materials Project (MP) database.[20] Chen et al. proposed a generalized MatErials Graph Network (MEGNet) for molecules and materials that was trained on 60,000 crystal structures from MP.[29] Park et al. developed an improved-CGCNN (iCGCNN)[28] with an alternative edge update method and trained on DFT formation enthalpy of 450,000 crystal structures in the Open Quantum Materials Database (OQMD).[22] The CGCNN and its variants exhibit similar accuracy in predicting formation enthalpy, with mean absolute error (MAE) of 0.03–0.04 eV/atom.[27–30]

For structure and stability predictions, it is imperative that the model is able to (1) predict the total energy of both GS and higher-energy structures with similar accuracy and (2) distinguish energetically favorable (low-energy) structures from those with higher energy. The CGCNN models discussed above are trained primarily on ICSD structures that are GS or near-GS structures. As we show in section "results and discussion," these models are likely to be biased toward GS structures and, therefore, inaccurate in predicting total energies of higher-energy structures. While the iCGCNN model[28] is trained on both GS and higher-energy structures, an explicit demonstration of the model performance for GS and higher-energy structures is missing. Since the focus of that study was to improve the overall prediction accuracy, it is not clear if the resulting model can, for a given composition, correctly rank the different structures based on their total energy.

In this work, we train our GNN model on a combined dataset consisting of both GS and higher-energy structures in a balanced fashion to accurately predict their total energy. We use DFT total energy of ~16,500 ICSD structures from the National Renewable Energy Laboratory (NREL) Materials

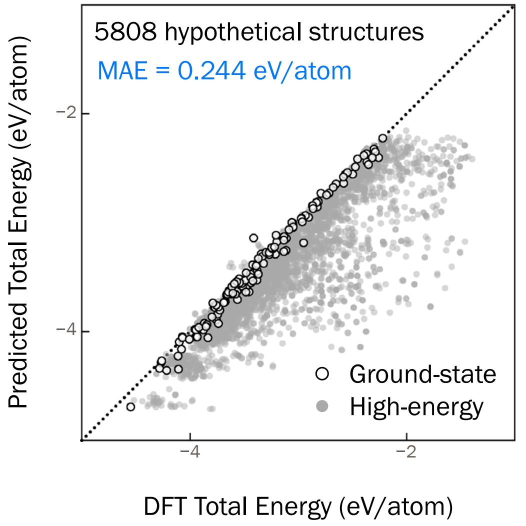

Figure 2. Energy underprediction of hypothetical structures

Total energy of hypothetical structures (see section "model trained on ICSD structures" for details) predicted with the ICSD model. The total energy is systematically underpredicted for the high-energy hypothetical structures suggesting model bias toward lower-energy structures.

We further confirm this bias by using the ICSD model to predict the total energy of ~5,800 hypothetical structures. As described in section "data and preparation" (experimental procedures), the dataset of hypothetical structures contains, in addition to the GS structures, a number of higher-energy hypothetical structures for a given composition. The ICSD model severely underpredicts the total energy of the higher-energy hypothetical structures but accurately predicts the energy of the corresponding GS structures (Figure 2), which highlights the model bias toward GS structures. For structure and stability predictions, a model that is accurate for both GS and higher-energy structures is desired.

Database[31] and ~11,000 hypothetical structures generated by the ionic substitution method.[32,33] While the overall prediction accuracy of our model is at par with other graph-based models (MAE = 0.04 eV/atom), with similar accuracy in predicting the total energy of GS *and* higher-energy hypothetical structures. We demonstrate the model's capability to satisfactorily distinguish low- and higher-energy structures for a given composition. Finally, we investigate the prediction outliers and find that, in some cases, the source of the error can be traced back to the inaccuracies in the DFT total energy.

RESULTS AND DISCUSSION

Model trained on ICSD structures

Previously reported GNN models for predicting total energy and formation enthalpy[27,29] were trained primarily on ICSD crystal structures with DFT total energy and formation enthalpy taken from the MP.[20] For benchmarking, we train a CGCNN model (Figure 9) on the DFT total energy of ICSD structures from the NREL Materials Database (NRELMatDB).[31] The model is trained on 15,500 crystal structures with 500 structures each withheld for validation and testing. We find that the prediction accuracy, gauged by the MAE, is 0.041 eV/atom (Figure 1A). The standard deviation in the MAE is ±0.005 eV/atom, which is obtained by training four different models and calculating the corresponding MAE on test sets each containing 500 crystal structures, with no overlap of structures between the test sets (Figure S1). The optimized hyperparameters for the model are provided in Table S1 of the supplemental information. Hereafter, we reference this model as the "ICSD model". The learning curve is presented in Figure 1C, which shows that at least 10^4 crystal structures are required to achieve a test MAE of <0.05 eV/atom, consistent with previous models.[27]

The formation enthalpy (ΔH_f) of a crystal structure with a chemical composition $A_xB_yC_z$ can be calculated from the DFT total energy as, $\Delta H_f = E_{total} - x\mu_A^0 - y\mu_B^0 - z\mu_C^0$, where E_{total} is DFT total energy of $A_xB_yC_z$ with ΔH_f and E_{total} expressed per formula unit and μ_i^0 are the reference chemical potentials of elements, typically under standard conditions. Since μ^0 are reference values, ΔH_f is linearly dependent on E_{total}. By design, the error in predicting ΔH_f is the same as in predicting total energy. The ICSD model has an MAE of 0.041 eV/atom for predicting DFT total energy. As such, ΔH_f can be predicted with the same accuracy, which is at par with other CGCNN models reported in the literature.[27–29] Furthermore, the typical experimental error in measuring formation enthalpy is the "chemical accuracy," which is on the order of 1 kcal/mol (0.043 eV/atom).[23] Assuming DFT calculated ΔH_f are reliable, the prediction error of the ICSD model is comparable with the chemical accuracy.

Figure 1B shows a histogram of the prediction errors relative to the DFT values, with 82% crystal structures (410 out of 500) predicted within an error of ±0.05 eV/atom. Of the remaining 90 structures lying outside the ±0.05 eV/atom error range, 51 structures are underpredicted, including PdN (space group #221) and CoMnP (space group #62), which are underpredicted by −0.733 eV/atom and −0.397 eV/atom, respectively. We find that these are higher-energy structures of those compositions reported in the ICSD, with PdN (space group #221) 0.459 eV/atom and CoMnP (space group #1) 0.400 eV/atom above the respective GS structures PdN (space group #225) and CoMnP (space group #62). Other underpredicted structures such as SiCN (space group #216) and AuN (space group #225) are highly unstable structures that lie above their respective convex hulls by 2.168 eV/atom and 1.897 eV/atom, respectively. The vast majority of ICSD structures have been determined through X-ray diffraction refinement of experimentally grown crystal structures with some metastable and computationally predicted hypothetical structures. As such, ICSD is biased toward stable, GS structures; the underprediction of the high-energy/unstable structures is a testament to this inherent bias, which so far has not been acknowledged in previous studies.[27–29]

Model trained on ICSD and hypothetical structures

To address the underestimation of the total energy of the hypothetical structures with the ICSD model, we first train a GNN model on the hypothetical structures separately (i.e., not including the ICSD structures). The training, validation, and test sets are chosen in a way to avoid overlap of compositions across them. For instance, all the hypothetical structures associated with the composition KGeP (*ABX* composition) appear only in the test set (Figure 3A) but not in the training or validation set. By avoiding overlap of compositions across the sets, we can eventually test the true performance of the model in energetically ranking the different structures associated with a given

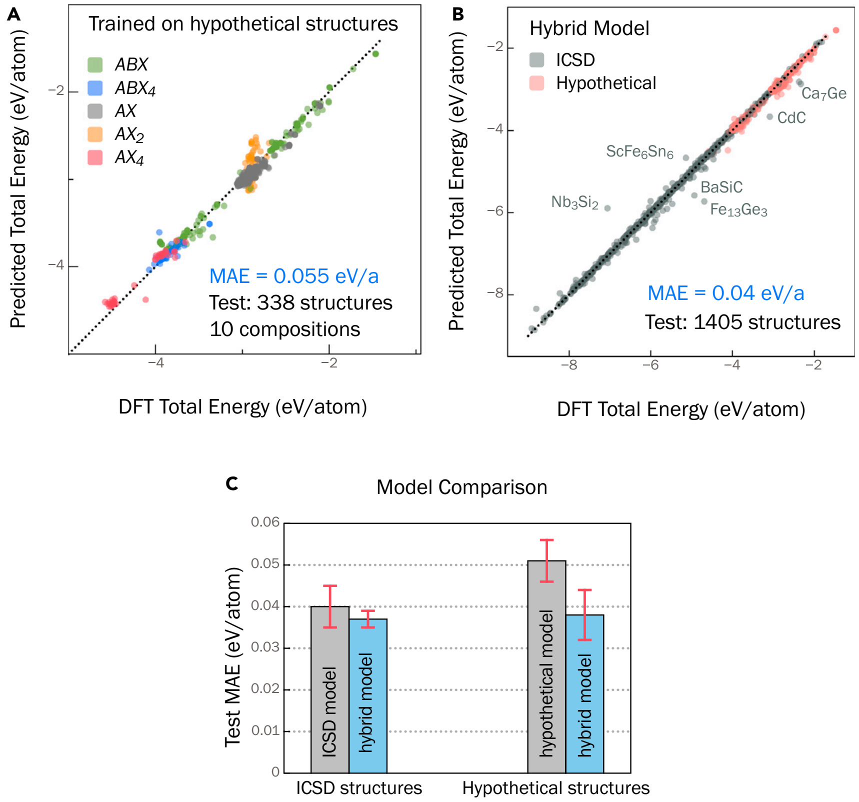

Figure 3. Model trained on combined dataset

(A) Predicted versus DFT total energy of the model trained only on hypothetical structures. The data points are colored by their composition type (see section "data and preparation" for details).

(B) Model trained on combined dataset of ICSD and hypothetical structures accurately predicts the total energy for both ICSD and hypothetical structures, with an overall MAE of 0.04 eV/atom.

(C) Comparison of predicted MAE for ICSD and hypothetical structures of the model trained only on ICSD structures (Figure 1A), model trained only on hypothetical structures shown in (A), and model trained on the combined dataset (blue). The standard deviation (shown as error bars) is calculated from four different models with non-overlapping test sets.

composition. In addition, at least one composition type (*ABX*, ABX_4, ...) is present in each of the sets.

First, the overall performance of this model with MAE = 0.055 eV/atom (Figure 3A) is significantly better than the performance of the ICSD model on the same structures (Figure 2). We find that the total energy of certain composition types, e.g., AX_2 (6 out of 191 compositions), that are under-represented in the hypothetical dataset are predicted with lower accuracy. In Figure 3A, the prediction outliers are predominantly of the AX_2 composition. Nonetheless, the overall performance is comparable with the ICSD model. However, when we use this model, trained on hypothetical structures only, to predict the total energy of 1,065 ICSD structures, we again find that the model performs poorly with an MAE = 0.424 eV/atom (Figure S2). As with the ICSD model (see section "model trained on ICSD structures"), this model again appears to be biased toward the hypothetical structures used in the training. To overcome this systematic bias, we find that it is practical to train a model on a combined dataset consisting of a balance between ICSD and hypothetical structures.

A GNN model is trained on a combined dataset of DFT total energy of 14,845 ICSD and 9,980 hypothetical structures (in 171 compositions) and validated and tested on 800 ICSD and ~600 hypothetical structures in 10 different compositions. An overall MAE of 0.04 eV/atom is achieved across ICSD and hypothetical structures (Figure 3B), which is comparable with the prediction accuracy of the ICSD model. The standard deviation in the MAE (0.005 eV/atom) is determined by training four different models and calculating the corresponding MAE on test sets each containing 800 ICSD and ~600 hypothetical structures (10 compositions each) with no overlap in the structures (Figure S3). The learning curve is presented in Figure S4, which shows that at least 2 × 10^4 crystal structures (twice as many as are required for the ICSD model) are required to achieve test MAE of <0.05 eV/atom. Figure 3C shows the individual MAEs for the ICSD and hypothetical structures. For comparison, the predicted MAE of the ICSD model (see section "model trained on ICSD structures") and the model trained on the hypothetical structures alone are provided. It is evident from Figure 3C that the model trained on the combined dataset improves the prediction accuracy for both ICSD and hypothetical structures and overcomes the model bias when each dataset is used separately to train a total energy model. We also train the MEGNet[29] and CGCNN[27] models on an identical combined dataset to demonstrate the generality of our choice of training data to alternative models. A comparison of the predicted MAE on ICSD and hypothetical structures is shown in Figure S5.

Energy ranking of structures

While it is crucial to have a high-accuracy model for predicting total energy, it remains to be seen whether the model can rank the different structures of a given composition in the correct order of their energies. As mentioned in section "introduction," this energy ranking is desired for distinguishing energetically favorable (low-energy) structures from the higher-energy unfavorable structures. Figure 4 shows the comparison between DFT and model-predicted relative total energy ($E - E_{min}$) of all the hypothetical structures for each of the 10 compositions present in the test set (Figure 3B). In general, our model-predicted energy rankings are in fair agreement with DFT, although there are noticeable differences depending on the composition type.

The rankings for the *ABX* type compositions (e.g., KGeP, KZnSb, and NaBeAs) are the most accurate: i.e., the model correctly identifies the GS structure and does not incorrectly misassign a higher-energy structure as low energy (Figure 4).

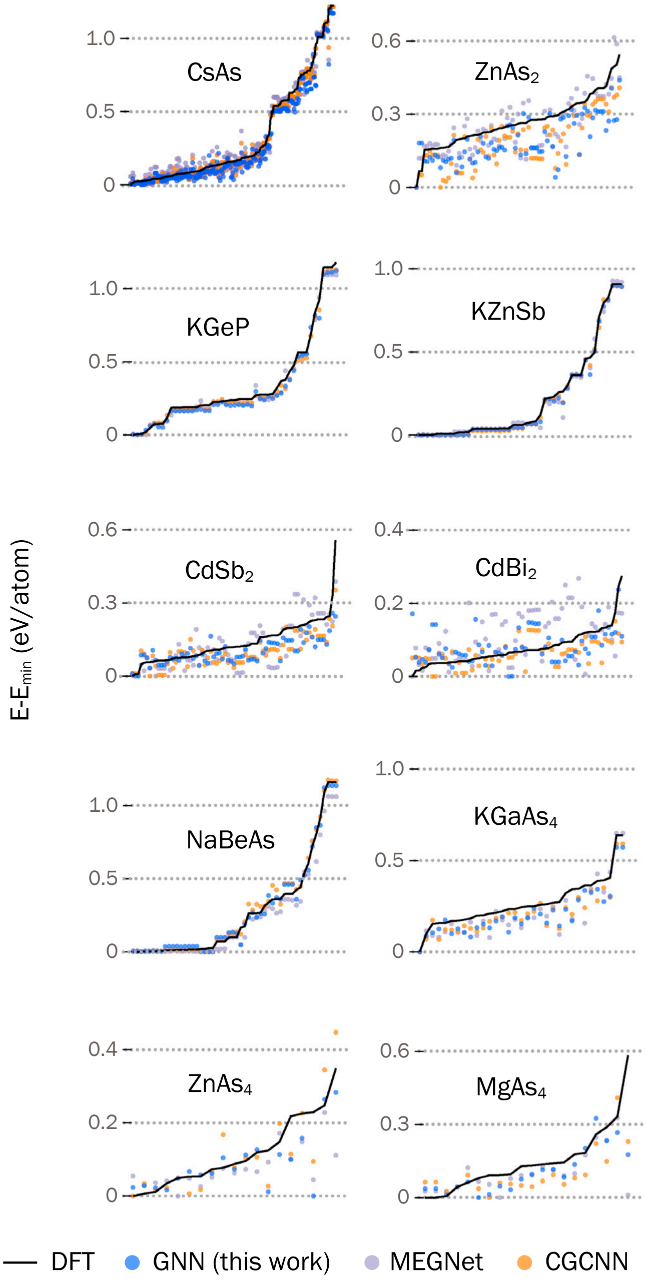

Figure 4. Energy ranking of hypothetical structures

Predicted relative energy ($E - E_{min}$) of hypothetical structures of 10 different compositions from the test set in Figure 3B compared with DFT. The x axes represent polymorphic structures, which are generated through ionic substitution.[32,33] For nine out of 10 compositions, the predicted GS either matches or is within 0.025 eV/atom of the DFT GS structure.

The good ranking of *ABX* composition type can be attributed to the fact that *ABX* comprises the largest fraction of the training dataset of hypothetical structures (139 out of 191 compositions). The ranking for CsAs (*AX* type composition) is satisfactory, with DFT GS structure predicted to be only 0.007 eV/atom higher than the GS structure predicted by the model. Moreover, none of the higher-energy structures are misassigned as low-energy structures. In the case of $KGaAs_4$, a ABX_4-type composition, the model correctly identifies the GS structure and also does not misassign any of the higher-energy structures as the GS.

On the other hand, for the AX_2 type compositions (e.g., $ZnAs_2$, $CdSb_2$, $CdBi_2$), the energy ranking of the structures requires a more detailed examination. The model correctly identifies the GS structure of $ZnAs_2$; however, a few high-energy structures are also identified as low energy. This energy ranking can be considered satisfactory because, in practical structure prediction implementations, one would consider a few lowest-energy structures as candidates for the GS structure. Similarly, the DFT GS structure of $CdSb_2$ is predicted to be only 0.009 eV/atom above the model-predicted GS, which will qualify the true GS structure as one of the lowest-energy structures. The model-predicted GS structure has a DFT relative energy ($E - E_{min}$) of 0.007 eV/atom. Finally, the energy ranking for $CdBi_2$ is inaccurate since the DFT GS structure is predicted to be 0.171 eV/atom above the model-predicted GS structure. It is evident from Figure 4 that the relative energies of all the $CdBi_2$ structures lie in a limited window of $\sim$0.25 eV/atom, unlike the *ABX*-, *AX*-, and ABX_4-type compositions. It is a more challenging to rank the structure in the correct order of their energies when all or a large fraction of the structures have similar energies: i.e., the energy differences cannot be sufficiently resolved.

For AX_4-type compositions, the energy rankings are similar to $ZnAs_2$ and $CdSb_2$, wherein the GS structures of $ZnAs_4$ and $MgAs_4$ are among the lowest-energy structures predicted by the model, with their DFT relative energies 0.023 eV/atom and 0.036 eV/atom, respectively. At the same time, a few high-energy structures are also identified as low energy.

While the model satisfactorily ranks the energies of hypothetical structures, we also inspect the rankings of known structures to establish the robustness of the model. We chose the known polymorphs of MgO and ZnO from the ICSD database as representative examples. Figure S6 shows the comparison between DFT and model (trained on combined dataset) predicted energy rankings. Out of the nine reported polymorphs of MgO, the model correctly labels the GS rock salt structure and also does not misassign the higher-energy structures as low energy. Similarly, out of the five reported polymorphs of ZnO, the model correctly labels the GS wurtzite structure and accurately ranks the higher-energy structures. In summary, the model satisfactorily ranks the energy of the structures for most of composition types. For nine out of 10 hypothetical compositions (Figure 4), the predicted GS structure either exactly matches or is within 0.025 eV/atom of the DFT GS structure.

We show the generality of our choice of training data to GNN models with similar architecture by training MEGNet[29] and CGCNN[27] models on identical training, validation, and test sets. The models trained on only ICSD structures consistently fail to rank the structures of a given composition in the correct order of their energies (Figure S7). The total energy of higher-energy structures is severely underestimated, which is expected due to the model bias toward low-energy structures (Figure S7), as discussed in section "model trained on ICSD and hypothetical structures." The models trained on a balanced combined dataset of GS and hypothetical structures overcome this limitation. Overall, the energy rankings predicted with our GNN model are similar to those predicted by MEGNet and CGCNN (Figure 4), when trained on the identical combined dataset.

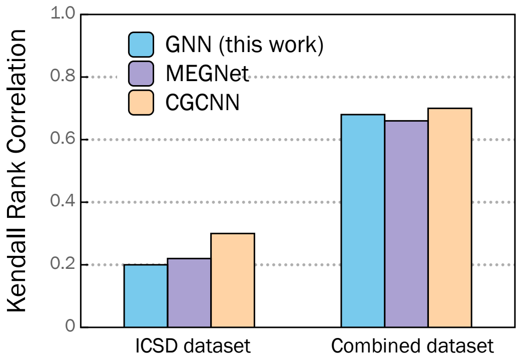

Figure 5. Comparison of energy rankings with different datasets and models

Comparison of KRCC, averaged over the 10 test compositions in Figure 4. Our GNN model, MEGNet, and CGCNN models, when trained on the identical combined dataset, perform similarly but significantly better than the same models trained on ICSD structures alone.

We use the Kendall rank correlation coefficient (KRCC) as a metric to compare the accuracy of the predicted energy rankings between different models and datasets. Higher accuracy in energy rankings corresponds to correlation coefficients close to +1. The KRCCs, averaged over the 10 test compositions (Figure 4), are compared in Figure 5. When trained on the identical combined dataset, our GNN model, MEGNet, and CGCNN models have similar average KRCCs. This is a significant improvement in KRCC compared with when the same models are trained on only ICSD structures (ICSD dataset versus combined dataset in Figure 5). Therefore, the choice of training data plays a more crucial role while the actual model architecture has a minor effect on the performance of the models in energy ranking of structures.

ML models based on kernel ridge regression[34] and random forest[35] methods have previously been trained on GS and high-energy structures to predict formation energy. Faber et al. developed a kernel ridge regression model to predict the formation energy of elpasolite ABC_2D_6 crystals, achieving an MAE of 0.1 eV/atom,[34] which is 2X-3X larger error compared with GNN models (0.03–0.04 eV/atom).[27,29] In the absence of an explicit demonstration, it is not clear whether this KRR model can accurately rank the polymorphic structures of a compound in the correct order of their energies. In contrast, the random forest model developed by Kim et al.[35] predicts the formation energy of quaternary $XX'YZ$ Heusler compounds with an MAE of 0.039 eV/atom. More importantly, they demonstrate a KRCC of 0.68, which is similar to the average KRCC with our GNN, MEGNet, and CGCNN models (Figure 5). The kernel ridge and random forest models were both trained for specific material families (ABC_2D_6, $XX'YZ$), which might limit their general applicability to other compositions. Perhaps, training these models on the combined dataset used in our work might result in similar performance across different compositions, but may require tedious feature engineering by hand, unlike in GNN models.

Analysis of prediction errors

We perform a thorough analysis of the large prediction errors in Figure 3B. Such an analysis is useful in attributing the error to either prediction outlier or inconsistency in the training data. The model trained on the combined dataset, presented in Figure 3B, predicts the total energy of ~79% (1,105 out of 1,405) structures with <0.05 eV/atom error. However, seven crystal structures (labeled in the figure) are either over- or underpredicted by 0.500 eV/atom, which are, interestingly, all ICSD structures. We analyze each of these structures on a case-by-case basis to understand the source of the error.

$Fe_{13}Ge_3$ (space group #221, ICSD: 150584) is severely underpredicted by 1.039 eV/atom relative to the DFT total energy. In this case, our analysis reveals that the DFT total energy is inaccurate. In magnetic compounds containing transition metals, the total energy is sensitive to the configuration of the magnetic moments.[36] $Fe_{13}Ge_3$ has a ferromagnetic GS; however, the DFT total energy in NRELMatDB is for the non-magnetic configuration. Upon recalculating the DFT total energy with ferromagnetic configuration, the prediction error is reduced to +0.08 eV/atom. This example highlights that DFT materials databases may contain occasional inconsistencies that can be flagged through ML regression.

The total energy of BaSiC (space group #107, ICSD: 168413) and CdC (space group #225, ICSD: 183177) are underpredicted by 0.651 eV/atom and 0.582 eV/atom, respectively. We find that both are hypothetical structures that were proposed in computational studies but not experimentally realized (ICSD contains a small fraction of hypothetical structures). These specific structures of BaSiC and CdC lie 0.795 eV/atom and 1.706 eV/atom above their respective convex hulls, which indicates that these high-energy structures are likely unstable. While the model is trained to predict the total energy of both GS and higher-energy structures, the training dataset of hypothetical structures spans 24 elements (see section "experimental procedures"), including Ba, Cd, and Si, but not C. The underprediction in the case of BaSiC and CdC is indicative of the remnant bias in the model toward lower-energy structures for compounds containing elements that are not in the hypothetical structure dataset.

The total energy for Ca_7Ge (space group #225, ICSD: 43321) is underpredicted by 0.545 eV/atom. Upon analyzing the crystal structure of this intermetallic compound, we find that the Ca–Ge bond lengths associated with the Ca(*4b*) Wyckoff site is 3.4 Å (Figure S8), which is significantly longer than typical Ca–Ge bond length (3 Å) in other Ca-Ge compounds (e.g., CaGe, Ca_2Ge, and Ca_5Ge_3). We perform a k-nearest neighbor (kNN) analysis on the penultimate site embeddings (see section "experimental procedures") to identify other structures in the training set with embeddings that resemble Ca_7Ge. The purpose of the kNN is to find a number of training samples closest in distance to a point in the test set. Principal component analysis (PCA) is first used to reduce the embedding space to 10 dimensions, and the 10 nearest neighbors for each site in Ca_7Ge are found from embeddings for sites in the training dataset. There are two unique Wyckoff sites of Ca (*4b*, *24d*) in Ca_7Ge; their 10 nearest neighbors are shown in Figure S8, which suggests that the *4b* site more resembles Sr and Ba (larger ionic radius than Ca), consistent with the long Ca–Ge bond lengths. This could also explain why Ca_7Ge is furthest from the convex hull (0.093 eV/atom) compared with other Ca-Ge structures.

CellPress
OPEN ACCESS

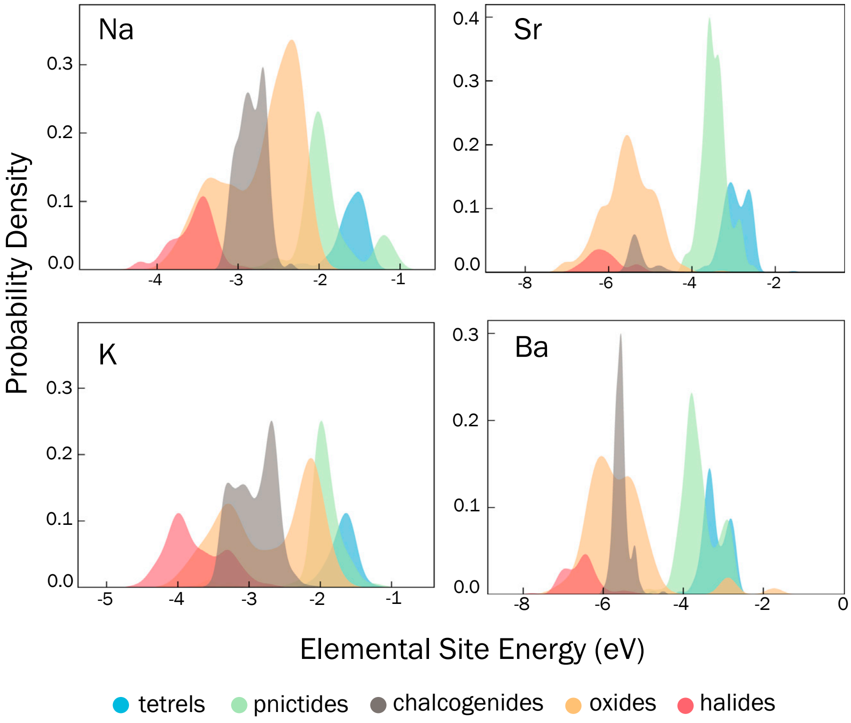

Figure 6. Elemental site energy distribution
Probability density of elemental site energies of Na, K, Sr, and Ba. The energy distribution provides chemical trends learned by the model. Generally, the site energies are more negative when the electropositive elements such as Na, K, Sr, and Ba are bonded to more electronegative anions (halides) than when bonded to less electronegative anions (pnictides, tetrels).

Another outlier, Nb_3Si_2 (space group #127, ICSD: 645431), is overpredicted by 1.163 eV/atom. In training the model, we directly use ICSD structures rather than DFT-relaxed structures. While, in most cases, the DFT-relaxed structures are not far from the ICSD structures, there are exceptions where this is not the case, such as for Nb_3Si_2. Using the DFT-relaxed structure instead of the ICSD structure reduces the error to 0.054 eV/atom.

Not all prediction errors are easily explainable as arising from the underlying DFT database. The source of error for $ScFe_6Sn_6$ (space group #191), which is overpredicted by 0.470 eV/atom, could not be identified and we believe that it is a case of prediction outlier. We thus have identified several causes of prediction errors, ranging from inconsistency in DFT data to simply model inaccuracy.

Chemical trends

Interpretability of predictive neural network models remains intrinsically challenging. While direct physical interpretation of the CGCNN model in this work may not be possible, we compare trends in the model predictions with general chemical principles. Specifically, we identify trends in the learned elemental site energies (see section "experimental procedures") through dimensionality reduction techniques such as PCA and t-distributed stochastic neighbor embedding (t-SNE). In conjunction, we also analyze the probability density of the elemental site energies.

We chose electropositive elements from group 1 (Na, K) and group 2 (Sr, Ba) as representative examples to identify trends in the learned elemental site energies. Figure 6 shows the probability density as a function of the elemental site energy for these elements. Figure 7 presents the corresponding two-dimensional t-SNE projections performed on the elemental embeddings. The site energy distributions in Figure 6 are calculated for all the sites in training set crystal structures for a given element. Only ICSD structures are considered in this analysis to avoid any unphysical effects arising from the hypothetical high-energy structures. For example, there are 1,095 unique Na-containing structures, with 7,056 unique Na Wyckoff sites. We find that when the element of interest is bonded to more electronegative anions—halogens (F, Cl, Br, I), O, or chalcogens (Se, Se, Te)—the resulting elemental site energies are more negative than when bonded only to less electronegative anions: tetrels (C, Si, Ge, Sn, Pb) or pnictogens (N, P, As, Sb, Bi). For example, out of 7,056 sites for Na, 3,458 sites bonded only to either halogens, O, or chalcogens span an energy range of [−4.36, −1.97] eV, whereas the 1,134 sites bonded only to tetrels or pnictogens span a lower energy range of [−3.17, −1.06] eV.

Notably the energy distributions for oxides span a wider energy range, overlapping with other anion types, which can be attributed to the large variety of oxide compositions and structures and the different cation coordinations. Generally, Na, K, Sr, and Ba prefer octahedral coordination (6-fold coordination) when bonded to O (e.g., rock salt Na_2O, BaO) but there can be a departure from this typical behavior depending on the presence of other cations. For instance, Na sites in $Na_{17}Al_5O_{16}$ (space group #8) and $Na_{14}Al_4O_{13}$ (space group #14) are 3-fold, 4-fold, and 5-fold coordinated with some of the elemental site energies lying in the "tail" of the oxides' (near the peak of pnictides) energy distribution (Figure S9). As such, some of the Na sites in these compounds behave as if they are bonded to pnictogens rather than O. The presence of Al, which generally prefers tetrahedral coordination, causes this departure from the typical behavior.

The t-SNE projections in Figure 7 offer an additional dimension (compared with the 1-D site energy distribution in Figure 6) to visualize the learned elemental distributions. The t-SNE projections reveal distinct clusters depending on the anion type, consistent with the observation of peaks in the probability density energy distributions (Figure 6). The separation into different clusters suggests that the chemical identity of the cation-anions bonds, at least for the four representative elements considered here, governs the learned elemental embedding. Consistent with the elemental site energy distribution, some Na sites in $Na_{17}Al_5O_{16}$ (space group #8) and $Na_{14}Al_4O_{13}$ (space group #14) lie in the cluster of pnictide embeddings (Figure S9).

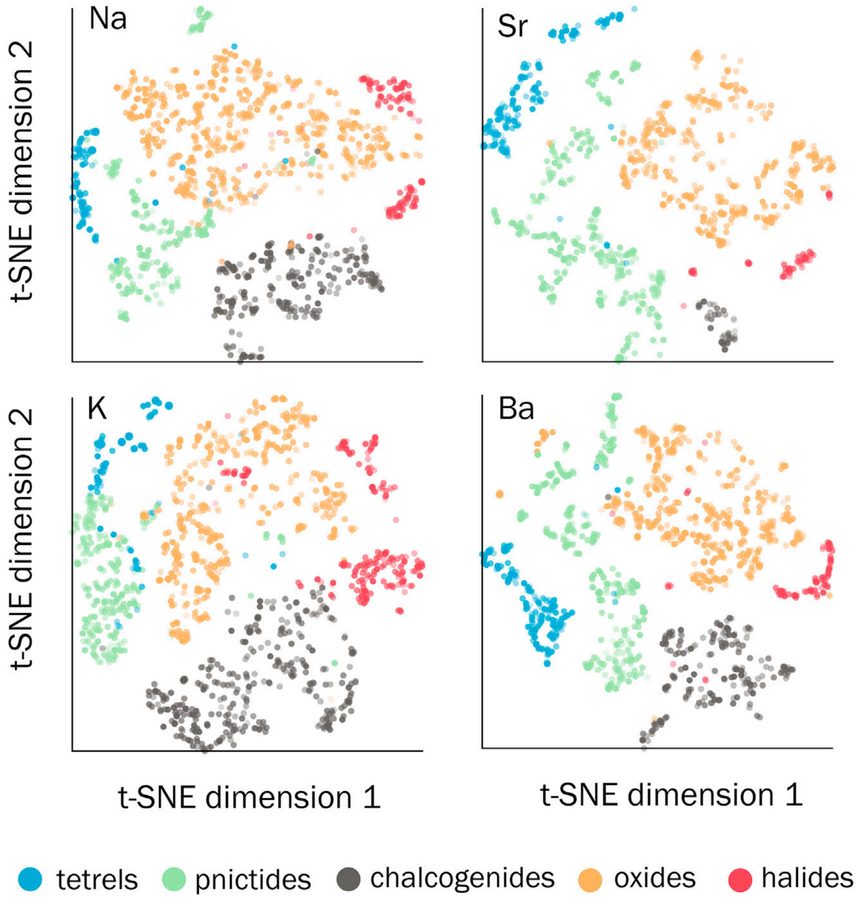

Figure 7. Clustering of elemental site energy
t-SNE visualizations of the PCA-reduced elemental embeddings of Na, K, Sr, and Ba, shown as representative examples. The training-set-extracted embeddings are analyzed to draw chemical trends learned by the model. The embeddings lie in four major clusters, depending on the local environment (oxides, chalcogenides, halides, pnictides, tetrels) of the element of interest.

Assessment of thermodynamic stability

Thermodynamic phase stability against decomposition into competing phases is a prerequisite for searching new materials and can be assessed through a convex hull construction.[23] Materials that lie on the convex hull are considered stable: i.e., the energy above the hull (ΔE_{hull}) is zero. Materials lying above the hull (ΔE_{hull}>0) are either unstable or metastable. The convex hull is defined as a convex envelope connecting the GS structures in a given chemical space and can be computed from DFT total energy by calculating formation enthalpy. For instance, in the binary Li-P chemical space, the convex hull connects elemental Li and P, and stable phases Li_3P, LiP, LiP_7, LiP_5, and Li_3P_7.

To demonstrate the accuracy of our GNN model in predicting thermodynamic phase stability, we perform convex hull analysis on a set of 1,794 ICSD compounds by using the model-predicted total energy of all the competing phases. Here, we consider all the competing phases documented in the ICSD. The ICSD compounds are chosen in the following way: all unique compounds present in NRELMatDB[31] formed by the 24 elements spanning group 1 (Li, Na), group 2 (Mg, Ca), group 3 (Sc), group 4 (Ti), group 5 (V), group 6 (Cr), group 7 (Mn), group 8 (Fe), group 9 (Co), group 10 (Ni), group 11 (Cu), group 12 (Zn), group 13 (B, Al), group 14 (C, Si), group 15 (N, P), group 16 (O, S), and group 17 (F, Cl). A total of 1,794 unique compounds (513 binary, 987 ternary, 288 quaternary, and 6 quinary) with 2–86 competing phases are identified.

ΔE_{hull} predicted with our GNN model is compared with the DFT values (for ΔE_{hull}< 1 eV/atom) for the 1,794 compounds in Figure 8. The comparison over the full energy range is provided in Figure S10. The stability predictions by the GNN model for 1,657 compounds (out of 1,794) are consistent with the DFT stability. The model correctly predicts 1,014 out of 1,078 stable compounds ($\Delta E_{hull} = 0$ eV in DFT). Among the 64 (out of 1,078) compounds that are incorrectly predicted to be unstable, 25 of them lie <0.005 eV/atom from the convex hull, which is well within the typical DFT error. Overall, our GNN-predicted phase stability is in fair agreement with DFT.

A precision-recall curve (PRC) provides a quantitative measure of the model's accuracy to classify a material as stable or unstable. Precision is defined as the ratio between the number of correctly classified stable materials (true-positive) and all materials classified as stable (true-positive + false-positive). Recall is the ratio between the number of correctly classified stable materials (true-positive) and all materials that are actually stable (true-positive + false-negative). We use the decomposition energies instead of ΔE_{hull} to determine the precision and recall scores for varying thresholds of decomposition energy. Here, decomposition energy is the minimum energy that the formation energy of an unstable material has to be lowered (more negative) before it becomes stable. Similarly, for a stable compound, we define the decomposition energy as the maximum energy that the formation energy can be increased (less negative) before it becomes unstable.[24] In this way, the decomposition energies of stable compounds are <0 eV/atom and for unstable compounds >0 eV/atom. The area under the PRC (AU-PRC) is 1 for perfect classification and 0 for random guess.

The AU-PRC of our GNN combined model tested on the 1,794 ICSD compounds is 0.98 (Figure S11). We find that the CGCNN model, when re-trained on our combined dataset, performs similarly in predicting thermodynamic stability (Figure S11B). We also perform phase stability analysis on the hypothetical structures to compare the performance of the ICSD and combined models. For this purpose, we consider the 10 hypothetical compositions (690 structures) from the test set of the combined model (Figure 4). Figure S12 shows a comparison of the predicted energy above the hull (ΔE_{hull}) with the ICSD only and combined models

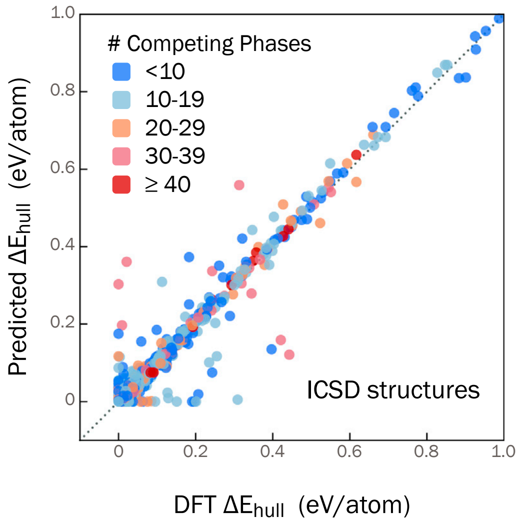

Figure 8. Prediction of thermodynamic stability

Comparison of energy above the convex hull (ΔE_{hull}) predicted with our GNN model (trained on the combined dataset) and with DFT for 1,794 ICSD compounds. The color scheme corresponds to the number of competing phases for each compound.

relative to the DFT calculated ΔE_{hull}. As expected, the ICSD model, which is biased toward GS structures, underpredicts ΔE_{hull} and, therefore, has a higher rate of false-positives (53%) in predicting thermodynamic stability. In contrast, the combined model has a much lower false-positive rate (1.5%). Consequently, the AU-PRC for the ICSD model (0.60) is much lower than for the combined model (0.99).

We also compare the stability predictions by our GNN model with those from simple composition-based models.[24] We train the Magpie model[37] on the lowest-energy structures for each composition in our dataset using 145 composition-based features. We test this model to predict the stability of a subset of 1,794 ICSD compounds (above); an AU-PRC of 0.78 (Figure S13) is obtained, which is significantly lower compared with our GNN model. The composition-based Magpie model is, as expected, biased toward GS structures and results in significantly more false-positives (36%) than the GNN model (8% false-positives).

Conclusions

In summary, we have developed a GNN model capable of reliably predicting DFT total energy of both GS and higher-energy structures. A model trained on a combined dataset consisting of a balance between both GS and higher-energy structures achieves a lower error than models trained on either GS or hypothetical structures alone. The accuracy of the resulting model is sufficient to rank the small differences in energy typically encountered between structures with the same composition. The model can, therefore, serve the purpose of rapidly screening the energetics of different configurations for a given composition, a critical step in elucidating the structure and stability of new chemistries.

Some of the large errors in energy predictions are explained by identifying their source of error as inconsistencies in the underlying training data. In small-scale DFT studies, each calculation can be carefully examined by the researcher to ensure convergence. In high-throughput DFT databases, however, manual analysis must be replaced with automatic convergence criteria that can occasionally miss peculiar cases. Therefore, the training and analysis of ML models is one way that the consistency of high-throughput DFT databases can be rapidly verified. ML predictions fail where the data are poorly explained by neighboring trends, either because insufficient similar examples exist, there are inconsistencies in the data, or there is extreme sensitivity of the regressed variable with respect to structure. In addition to highlighting data inconsistencies and where additional data should be collected, prediction outliers can highlight interesting and unique chemical functionality that might otherwise go unnoticed in large databases.

There are a few limitations to the model, which remain to be addressed. The hypothetical structures used for training the model span only 24 elements, and their total energy is confined to a small range, in contrast to the wide range in the total energy of ICSD structures. To overcome this limitation, generation of additional DFT data for hypothetical structures will be done in a future work. Additionally, the current model was trained on hypothetical structures after DFT relaxations, which limits its usefulness in the forward screening of new hypothetical structures, where relaxed coordinates are not available. Generating accurate predictions with unrelaxed structures remains an unresolved problem in the field of structure prediction.

EXPERIMENTAL PROCEDURES

Resource availability

Lead contact

The lead contact is Prashun Gorai (pgorai@mines.edu).

Materials availability

There are no physical samples generated in this work.

Data and code availability

A frozen version of the code is available on Zenodo (https://doi.org/10.5281/zenodo.5484194) and in a GitHub repository (https://github.com/prashungorai/combined-gnn).

GNN architecture

A CGCNN was constructed as depicted in Figure 9. Crystal structures are first converted to a graph using pymatgen,[38] using atomic sites as the graph nodes and distances between sites as the graph edges. Each node in the graph has exactly 12 edges, corresponding to the 12 nearest neighbor sites in the crystal while accounting for periodic boundaries. Node features include only the identity of the element at the atomic site, and edge features only included the raw distances (in angstroms) between the two sites. This is in contrast to other CGCNN models[27–29] that use several additional node and edge features: e.g., group and period number, electronegativity. An embedding layer is used to convert the discrete element type of each atomic site into a 256-parameter vector, functioning similarly to a one-hot encoding of the atom type followed by a dense layer of dimension 256. Edge features are initialized from the raw distances through a radial basis function expansion, $r_i(d) = \exp[-\eta(d - c_i)]$ for $i \in [1, \ldots, 10]$, where d is the edge distance and η, c_i are learned parameters initialized to 7 and $[0, 0.7, 1.4, 2.1, \ldots, 6.3]$, respectively. In the CGCNN, the node and edge features are updated by passing them through a series of message layers, in which the nodes exchange information with their neighboring edges.

The structure of the message-passing layers is adapted from Jørgensen et al.[39] First, for each edge, the source and target site features are concatenated with the edge's features, passed through a series of dense layers, and added to the input edge features in a residual fashion. Next, node

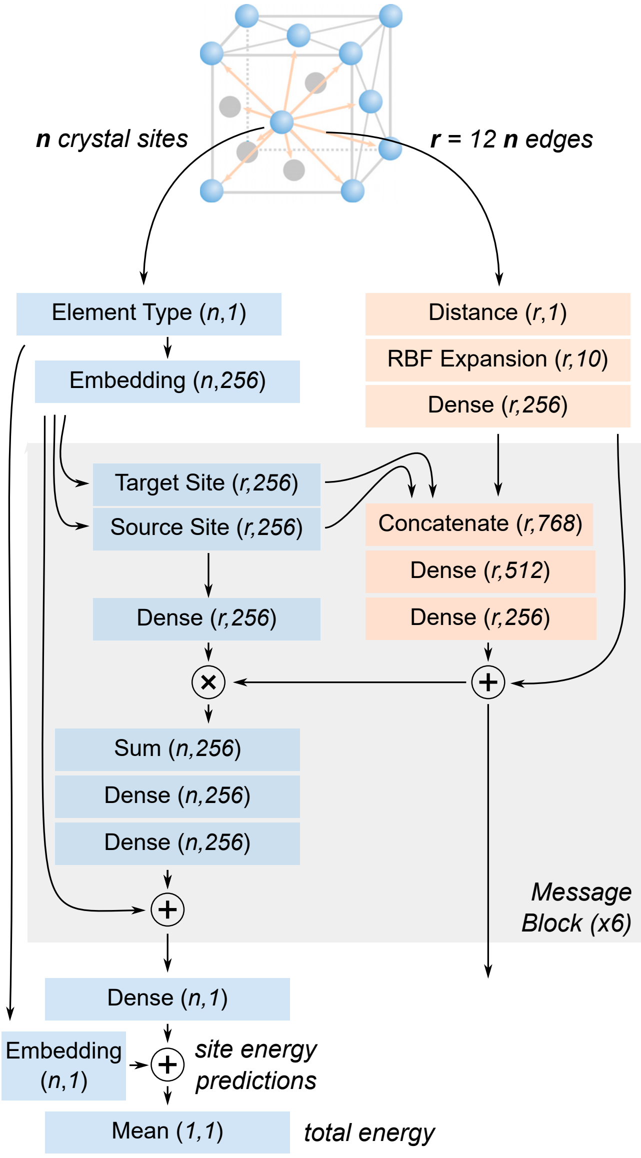

Figure 9. GNN model architecture

Schematic of the neural network architecture. Node (atomic sites) and edge features (interatomic distances) output from each message block are fed as inputs into the subsequent block. The model predicts energy per site for all the sites in a given structure, which are averaged to get the total energy.

features are updated using the features of the neighboring sites and those of the connecting edges. For each of the 12 edges pointing into a given site, the feature vectors of the source sites are multiplied by features of the corresponding edge before all 12 vectors are summed together. The resulting feature vector is then passed through a series of dense layers before being added to the original site feature vector in a residual fashion. Outputs from each message block are then fed as inputs into a subsequent message block for a total of six message layers. Final total energy predictions are produced by feeding the final site features into a 1-D output layer, producing a single energy prediction for each site. These predictions are added to a learnable mean energy for each element before being averaged over all sites in the crystal to produce a mean energy prediction.[40] Site-level contributions to the total predicted energy can therefore be extracted from this penultimate layer.

CGCNNs are trained for 500 epochs over the training data with a batch size of 64 crystals using the Adam optimizer with weight decay. The learning rate was decayed starting from an initial value of $1e^{-3}$, according to $1e^{-3}/(1+\text{epoch}/50)$, and the weight decay was similarly decayed according to $1e^{-5}/(1+\text{epoch}/50)$. The loss function minimized was the MAE between predicted and DFT total energy.

Data and preparation

Three distinct datasets of DFT-computed total energy are used in training the CGCNN models. First, we use DFT total energy of ~14,000 ordered and stoichiometric crystal structures from the ICSD[8] that are available in the NREL Materials Database (NRELMatDB).[31] The DFT calculations are performed with VASP[41]; details of the calculations are available from Stevanović et al.[23]

During data cleanup, we identified that the DFT calculations for 1,677 structures containing fluorine were insufficiently converged. We recalculated the DFT total energy of 874 (out of the 1,677) structures with a recommended larger plane-wave energy cutoff of 540 eV. The remaining 803 structures contain transition elements that require an exhaustive search of the different magnetic configurations to determine the GS structure. Given the high computational cost associated with the search for the magnetic GS configurations, these 803 structures were not recalculated and also not included in the training data. With future applications of our CGCNN model in mind, we expanded the dataset to including DFT total energy of ~3,900 ICSD structures containing mixed anions (e.g., ZrOS), which are not currently in NRELMatDB. The DFT methodology (GGA-PBE)[42] and calculation parameters for the mixed-anion compounds are consistent with those used in NRELMatDB. Combined, we use DFT total energy of ~16,500 ICSD structures to train, validate, and test the CGCNN models. The ICSD collection identifiers along with their total energy are made available through a public GitHub repository.[43] This dataset of ICSD structures spans 60 elements and 12,760 unique compositions, with 2,113 compositions existing in more than one structure.

We also leverage a dataset of ~11,000 hypothetical structures that were created by ionic substitutions in known prototype structures from the ICSD.[33,32] Upon ionic substitution, the decorated structures are relaxed and their total energy is calculated with DFT. The relaxed structures (as VASP POSCAR files) and the total energy are available through the GitHub repository.[43] The dataset is created for the purpose of discovering new Zintl phases.[32,33] As such, it spans 24 elements in 191 unique compositions of the type *ABX* (139, 6,087), AX_4 (18, 318), *AX* (15, 3,775), ABX_4 (13, 410), and AX_2 (6, 444), where the first number in parentheses is the number of compositions and the second number is the number of structures. Here, element *A* includes Li, Na, K, Rb, Cs, Ba, Mg, Sr, Zn, Cd; element *B* are Si, Ge, Sn, Pb, Zn, Cd, and Be; and *X* are group 15 elements (pnictogens) such as P, As, Sb, and Bi. KSnSb, $MgAs_4$, CdSb, $KGaSb_4$, and $ZnAs_2$ are representative compositions from this hypothetical structure dataset.

Analysis of atomic site energy

The learned elemental site energies (Figure 6), which are the site-level contributions to the total energy, are analyzed to identify chemical trends. For specific elements, we calculate the probability density of the atomic site energies from all the ICSD structures in the dataset. We do not include the hypothetical high-energy structures in the analysis of the site energies to avoid biasing the chemical trends toward unstable structures. The distribution of pairwise distances between the learned elemental embeddings (Figure 7) will encode the relation between materials. We utilize common dimensionality reduction techniques such as PCA[44] and t-SNE,[45] as implemented in scikit-learn,[46] to analyze the multi-dimensional elemental embeddings.

SUPPLEMENTAL INFORMATION

Supplemental information can be found online at https://doi.org/10.1016/j.patter.2021.100361.

ACKNOWLEDGMENTS

Funding for this work is provided by the US Department of Energy (DOE) Advanced Research Projects Agency – Energy (ARPA-E) program. S.P.,

CellPress
OPEN ACCESS

V.S., and P.G. acknowledge funding from ARPA-E under award number DE-AR0001205. P.S.J. acknowledges funding from ARPA-E under award number 19/CJ000/07/02. P.G. also acknowledges support from NSF through award DMR-2102409. J.Q. is funded by the NSF DIGI-MAT program, grant no. 1922758. This work was authored in part by the NREL, operated by the Alliance for Sustainable Energy, LLC, for the DOE under contract no. DE-AC36-08GO28308. The views expressed in the article do not necessarily represent the views of the DOE or the US Government. The research was performed using computational resources sponsored by the DOE's Office of Energy Efficiency and Renewable Energy (EERE) and located at NREL.

AUTHOR CONTRIBUTIONS

P.G. and P.S.J. conceived and designed this research project. S.P. and P.S.J. built and trained the neural network models. J.Q. and P.G. curated the training data. S.P., J.Q., and P.G. performed the first-principles DFT calculations. S.P., V.S., P.S.J., and P.G. analyzed the results. All authors participated in preparing and editing the manuscript.

DECLARATION OF INTERESTS

The authors declare no competing interests.

Received: April 15, 2021
Revised: July 31, 2021
Accepted: September 9, 2021
Published: September 30, 2021

REFERENCES

1. Janet, J.P., Liu, F., Nandy, A., Duan, C., Yang, T., Lin, S., et al. (2019). Designing in the face of uncertainty: exploiting electronic structure and machine learning models for discovery in inorganic chemistry. Inorg. Chem. *58*, 10592–10606.
2. Alberi, K., Nardelli, M.B., Zakutayev, A., Mitas, L., Curtarolo, S., Jain, A., Fornari, M., Marzari, N., Takeuchi, I., Green, M.L., et al. (2018). The 2019 materials by design roadmap. J. Phys. D Appl. Phys. *52*, 013001 1–48.
3. A.R. Oganov, G. Saleh, and A.G. Kvashnin, eds. (2019). Computational Materials Discovery (The Royal Society of Chemistry).
4. Curtarolo, S., Hart, G.L., Nardelli, M.B., Mingo, N., Sanvito, S., and Levy, O. (2013). The high-throughput highway to computational materials design. Nat. Mater. *12*, 191–201.
5. Hautier, G., Jain, A., and Ong, S.P. (2012). From the computer to the laboratory: materials discovery and design using first-principles calculations. J. Mater. Sci. *47*, 7317–7340.
6. Jain, A., Shin, Y., and Persson, K.A. (2016). Computational predictions of energy materials using density functional theory. Nat. Rev. Mater. *1-13*, 1.
7. Gorai, P., Stevanovic, V., and Toberer, E.S. (2017). Computationally guided discovery of thermoelectric materials. Nat. Rev. Mater. *2*, 1–16.
8. Belsky, A., Hellenbrandt, M., Karen, V.L., and Luksch, P. (2002). New developments in the Inorganic Crystal Structure Database (ICSD): accessibility in support of materials research and design. Acta Crystallogr. Section B *58*, 364–369.
9. Davies, D.W., Butler, K.T., Jackson, A.J., Morris, A., Frost, J.M., Skelton, J.M., and Walsh, A. (2016). Computational screening of all stoichiometric inorganic materials. Chem *1*, 617–627.
10. Doll, K., Schön, J.C., and Jansen, M. (2008). Structure prediction based on ab initio simulated annealing for boron nitride. Phys. Rev. B *78*, 144110.
11. Woodley, M., Battle, S.D., Gale, P.D., Richard, A., and Catlow, C. (1999). The prediction of inorganic crystal structures using a genetic algorithm and energy minimisation. Phys. Chem. Chem. Phys. *1*, 2535–2542.
12. Pickard, C.J., and Needs, R.J. (2011). Ab initio random structure searching. J. Phys. Condens Matter. *23*, 053201.
13. Stevanovic, V. (2016). Sampling polymorphs of ionic solids using random superlattices. Phys. Rev. Lett. *116*, 075503.
14. Gautier, R., Zhang, X., Hu, L., Yu, L., Lin, Y., Sunde, T.O.L., Chon, D., Poeppelmeier, K.R., and Zunger, A. (2015). Prediction and accelerated laboratory discovery of previously unknown 18-electron ABX compounds. Nat. Chem. *7*, 308–316.
15. Zhang, X., Yu, L., Zakutayev, A., and Zunger, A. (2012). Sorting stable versus unstable hypothetical compounds: the case of multi-functional ABX half-Heusler filled tetrahedral structures. Adv. Func Mater. *22*, 1425–1435.
16. Hautier, G., Fischer, C., Ehrlacher, V., Jain, A., and Ceder, G. (2011). Data mined ionic substitutions for the discovery of new compounds. Inorg. Chem. *50*, 656–663.
17. Balachandran, P.V., Young, J., Lookman, T., and Rondinelli, J.M. (2017). Learning from data to design functional materials without inversion symmetry. Nat. Comm. *8*, 1–13.
18. Chmiela, S., Tkatchenko, A., Sauceda, H.E., Poltavsky, I., Schütt, K.T., and Müller, K.R. (2017). Machine learning of accurate energy-conserving molecular force fields. Sci. Adv. *3*, e1603015.
19. Sauceda, H.E., Gastegger, M., Chmiela, S., Müller, K.R., and Tkatchenko, A. (2020). Molecular force fields with gradient-domain machine learning (GDML): comparison and synergies with classical force fields. J. Chem. Phys. *153*, 124109.
20. Jain, A., Ong, S.P., Hautier, G., Chen, W., Richards, W.D., Dacek, S., Cholia, S., Gunter, D., Skinner, D., Ceder, G., and Persson, K.A. (2013). Commentary: the materials project: a materials genome approach to accelerating materials innovation. APL Mater. *1*, 011002.
21. Curtarolo, S., Setyawan, W., Hart, G.L.W., Jahnatek, M., Chepulskii, R.V., Taylor, R.H., Wang, S., Xue, J., Yang, K., Levy, O., et al. (2012). Aflow: an automatic framework for high-throughput materials discovery. Comp. Mater. Sci. *58*, 218–226.
22. Kirklin, S., Saal, J.E., Meredig, B., Thompson, A., Doak, J.W., Muratahan, A., Rühl, S., and Wolverton, C. (2015). The open quantum materials database (OQMD): assessing the accuracy of DFT formation energies. Npj Comput. Mater. *1-15*, 15010.
23. Stevanović, V., Lany, S., Zhang, X., and Zunger, A. (2012). Correcting density functional theory for accurate predictions of compound enthalpies of formation: fitted elemental-phase reference energies. Phys. Rev. B *85*, 115104.
24. Bartel, C., Trewartha, A., Wang, Q., Dunn, A., Jain, A., and Ceder, G. (2020). A critical examination of compound stability predictions from machine-learned formation energies. NPJ Comput. Mater. *6*, 1–11.
25. Schmidt, J., Marques, M., Botti, S., and Marques, M.A.L. (2019). Recent advances and applications of machine learning in solid-state materials science. NPJ Comput. Mater. *5*, 1–36.
26. Deml, A.M., O'Hayre, R., Wolverton, C., and Stevanović, V. (2016). Predicting density functional theory total energies and enthalpies of formation of metal-nonmetal compounds by linear regression. Phys. Rev. B *93*, 085142.
27. Xie, T., and Grossman, J.C. (2018). Crystal graph convolutional neural networks for an accurate and interpretable prediction of material properties. Phys. Rev. Lett. *120*, 145301.
28. Park, C.W., and Wolverton, C. (2020). Developing an improved crystal graph convolutional neural network framework for accelerated materials discovery. Phys. Rev. Mater. *4*, 063801.
29. Chen, C., Ye, W., Zuo, Y., Zheng, C., and Ong, S.P. (2019). Graph networks as a universal machine learning framework for molecules and crystals. Chem. Mater. *31*, 3564–3572.
30. Chen, C., Zuo, Y., Ye, W., Li, X., and Ong, S.P. (2021). Learning properties of ordered and disordered materials from multi-fidelity data. Nat. Comp. Sci. *1*, 46–53.
31. NRELMatDB: NREL Materials Database. materials.nrel.gov.
32. Qu, J., Stevanovic, V., Ertekin, E., and Gorai, P. (2020). Doping by design: finding new n-type dopable abx_4 zintl phases for thermoelectrics. J. Mater. Chem. A *8*, 25306–25315.

33. Gorai, P., Ganose, A.M., Faghaninia, A., Jain, A., and Stevanovic, V. (2020). Computational discovery of promising new *n*-type dopable ABX zintl thermoelectric materials. Mater. Horiz. *7*, 1809–1818.

34. Faber, F.A., Lindmaa, A., von Lilienfeld, O.A., and Armiento, R. (2016). Machine learning energies of 2 million elpasolite $(abc_2D_6)(\boldsymbol{abC_2D_6})$ crystals. Phys. Rev. Lett. *117*, 135502.

35. Kim, K., Ward, L., He, J., Krishna, A., Agrawal, A., and Wolverton, C. (2018). Machine-learning-accelerated high-throughput materials screening: discovery of novel quaternary Heusler compounds. Phys. Rev. Mater. *2*, 123801.

36. Gorai, P., Toberer, E.S., and Stevanovic, V. (2016). Thermoelectricity in transition metal compounds: the role of spin disorder. Phys. Chem. Chem. Phys. *18*, 31777–31786.

37. Ward, L., Agrawal, A., Choudhary, A., and Wolverton, C. (2016). A general-purpose machine learning framework for predicting properties of inorganic materials. NPJ Comput. Mater. *2*, 1–7.

38. Ong, S.P., Richards, W.D., Jain, A., Hautier, G., Kocher, M., Cholia, S., Gunter, D., Chevrier, V.L., Persson, K.A., and Ceder, G. (2013). Python materials genomics (pymatgen): a robust, open-source python library for materials analysis. Comput. Mater. Sci. *68*, 314–319.

39. Jørgensen, P.B., Jacobsen, K.W., and Schmidt, M.N. (2018). Neural message passing with edge updates for predicting properties of molecules and materials. arXiv, 180603146, arXiv:1806.03146.

40. Schütt, K.T., Sauceda, H.E., Kindermans, P.J., Tkatchenko, A., and Müller, K.R. (2018). Schnet – a deep learning architecture for molecules and materials. J. Chem. Phys. *148*, 241722.

41. Kresse, G., and Furthmüller, J. (1996). Efficient iterative schemes for ab initio total-energy calculations using a plane-wave basis set. Phys. Rev. B *54*, 11169.

42. Perdew, J.P., Burke, K., and Ernzerhof, M. (1996). Generalized gradient approximation made simple. Phys. Rev. Lett. *77*, 3865.

43. Pandey, S., Qu, J., Stevanovic, V., St. John, P., and Gorai, P. (2021). GNN For Predicting Energy of Known and Hypothetical Structures. https://github.com/prashungorai/combined-gnn.

44. Hotelling, H. (1993). Analysis of a complex of statistical variables into principal components. J. Educ. Psychol. *6*, 417–441.

45. van der Maaten, L., and Hinton, G. (2008). Visualizing data using t-SNE. J. Mach. Learn. Res. *9*, 2579.

46. Pedregosa, F., Varoquaux, G., Gramfort, A., Michel, V., Thirion, B., Grisel, O., Blondel, M., Prettenhofer, P., Weiss, R., Dubourg, V., et al. (2011). Scikit-learn: machine learning in python. J. Mach. Learn Res. *12*, 2825–2830.

Patterns, Volume *2*

Supplemental information

Predicting energy and stability of known and hypothetical crystals using graph neural network

Shubham Pandey, Jiaxing Qu, Vladan Stevanović, Peter St. John, and Prashun Gorai

Supplemental Experimental Procedures

1. Optimized Hyperparameters

The hyperparameters include parameters used to generate the crystal graphs, parameters of the neural networks, and parameters that control the training process. The hyperparameters are optimized through a train-validation process, on a fixed validation set. The following ranges of hyperparameters are searched: (1) batch size: 32–64, (2) embedding dimensions: 64–256, (3) number of message blocks: 4-8, and (4) learning rate: $1e^{-n}$, $n = 3$-5. The mean absolute error of total energy prediction is reduced by 0.005 eV/atom by using a weight decay compared to when not using it.

Table S1: List of optimized hyperparameters in this work

Hyperparameter	Optimized value
Batch size	64
Embedding dimension	256
Number of message blocks	6
Learning rate	$1e^{-3}$
Weight decay	$1e^{-5}$
Number of epochs	500

2. Performance of the Models Trained on Total Energy of ICSD Structures

To estimate the uncertainty in the mean absolute error (MAE) of total energy prediction, four different models are trained on the DFT total energy of ICSD structures. The uncertainty in the MAE is the standard deviation across the four models, each tested on a different hold-out test set.

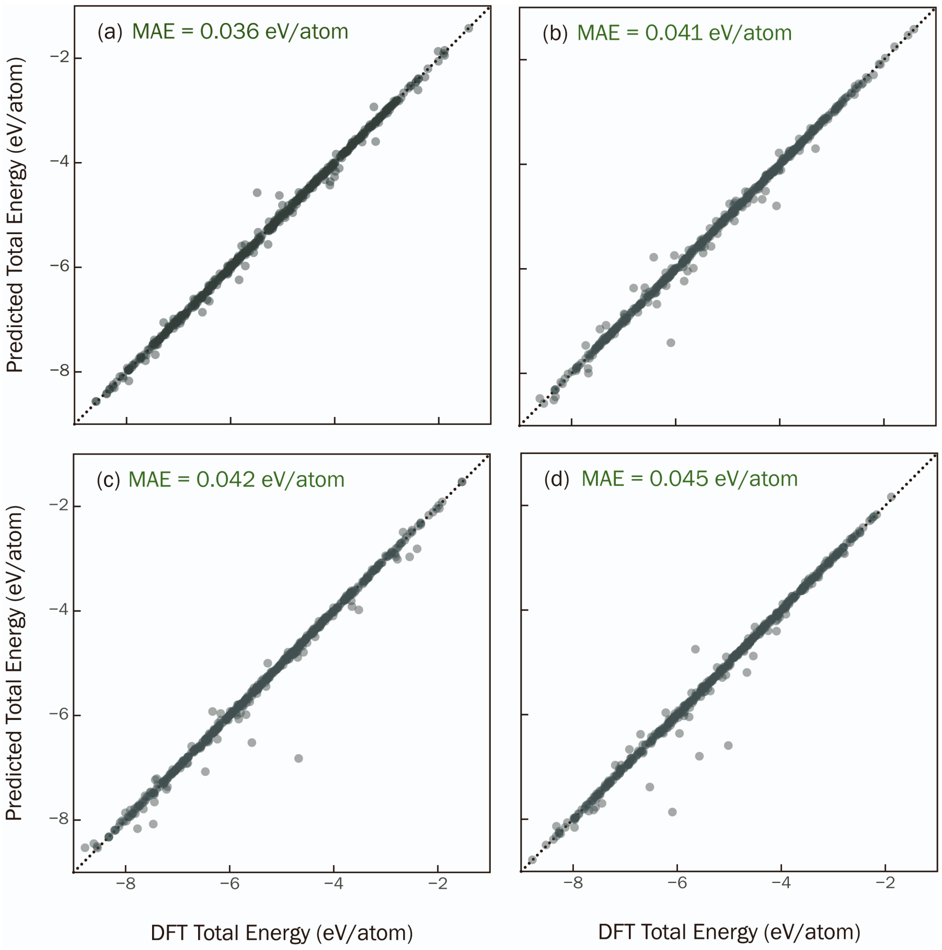

Figure S1: **Models trained on ICSD structures:** Convolutional neural networks trained on DFT total energy of ICSD structures from the NREL Materials Database. (a)-(d) Performance of the models trained and tested on four different sets of crystal structures. The mean absolute errors (MAEs) for the four different test sets are (a) 0.036 eV/atom, (b) 0.041 eV/atom, (c) 0.042 eV/atom, and (d) 0.045 eV/atom. The overall MAE across the four models is 0.041±0.005 eV/atom.

3. Performance of Model Trained on Total Energy of Hypothetical Structures

The model trained exclusively on the hypothetical structures is used to predict the total energy of the ICSD structures. Only a subset of ICSD structures, which contain the same 24 elements present in the hypothetical structure dataset, are chosen. The model poorly predicts the total energy of the ICSD structures with a large mean absolute error.

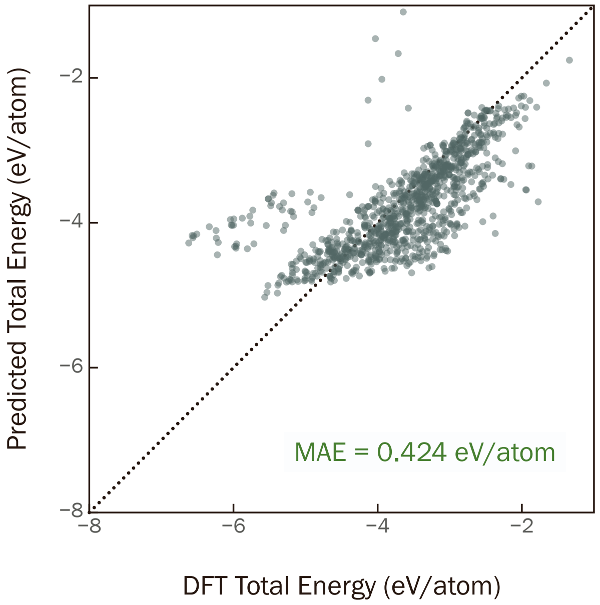

Figure S2: **Model trained on hypothetical structures:** The total energy of 1065 ICSD structures is predicted with the model trained on the hypothetical structures alone (see Section 2.2 in the main text). The predicted total energy has large errors compared to the DFT values. The mean absolute error (MAE) of the test set prediction is 0.424 eV/atom, suggesting the model is biased towards hypothetical structures.

4. Performance of the Hybrid Model Trained on Total Energy of ICSD and Hypothetical Structures

To estimate the uncertainty in the mean absolute error (MAE) of total energy prediction, four different models are trained on the DFT total energy of ICSD and hypothetical structures. The uncertainty in the MAE is the standard deviation across the four models, each tested on a different hold-out test set. The training, validation and test sets are chosen with no overlap of compositions for the hypothetical structures.

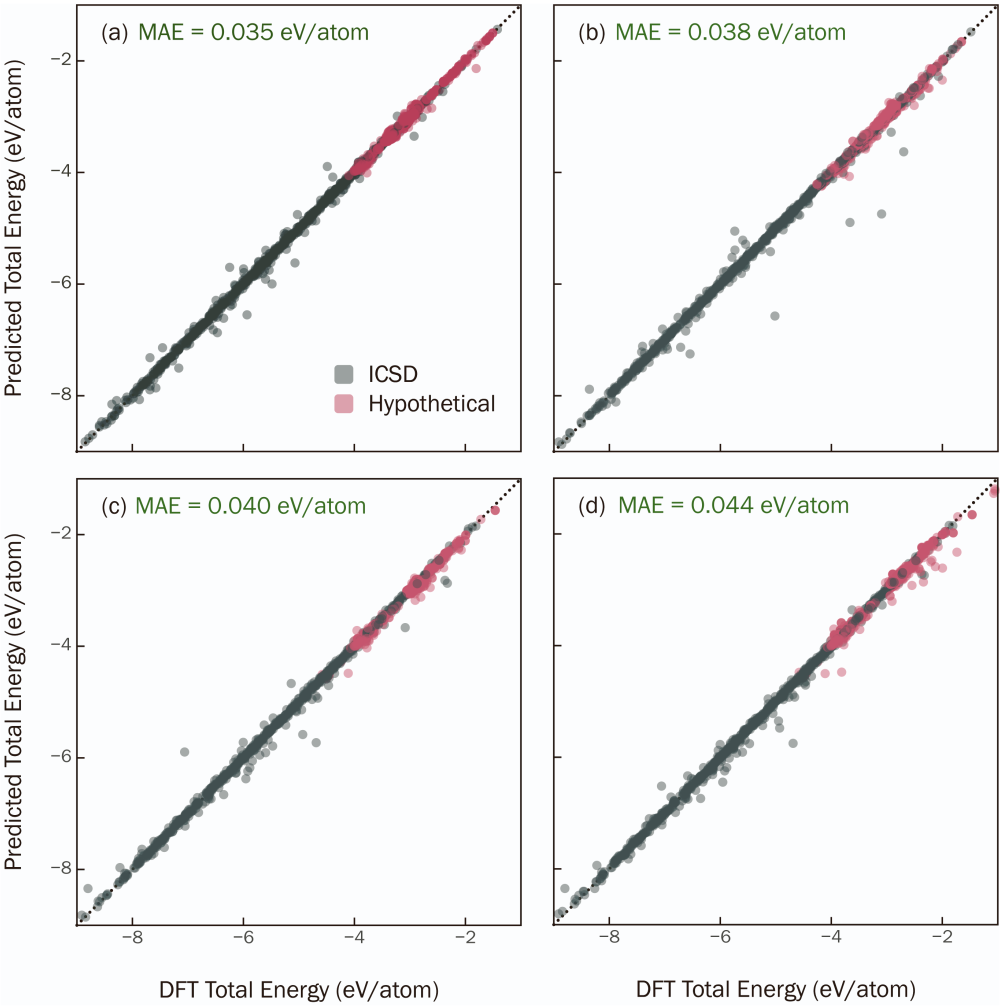

Figure S3: **Models trained on combined dataset:** Convolutional neural networks trained on DFT total energy of ICSD and hypothetical structures. (a)-(d) Performance of the models trained and tested on four different sets of crystal structures. The mean absolute errors (MAEs) for the four different test sets are (a) 0.035 eV/atom, (b) 0.038 eV/atom, (c) 0.040 eV/atom, and (d) 0.044 eV/atom. Gray(red) data-points correspond to ICSD(hypothetical) structures The overall MAE across the four models is 0.040±0.005 eV/atom.

5. Learning Curve of the Model Trained on ICSD and Hypothetical Structures

A learning curve compares the performance of a model on a test set for varying number of training instances and therefore, can provide insights into whether a model is overfitted. The learning curve for the model trained on the "combined" dataset of ICSD anf hypothetical structures shows that: (1) there is a systematic improvement in the model performance with the number of training crystal structures, and (2) the minimum number of training structures to achieve an MAE<0.05 eV/atom is $\sim 2 \times 10^4$.

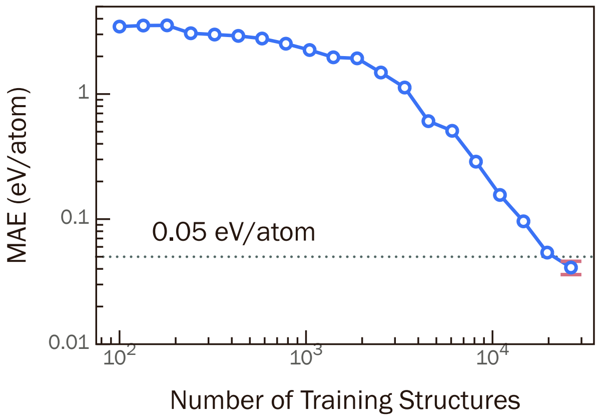

Figure S4: **Learning curve of model trained on combined dataset:** Learning curve for the hybrid model, showing that at least $2{\times}10^4$ crystal structures are required to achieve an MAE of <0.05 eV/atom.

6. Comparison of Prediction Accuracy

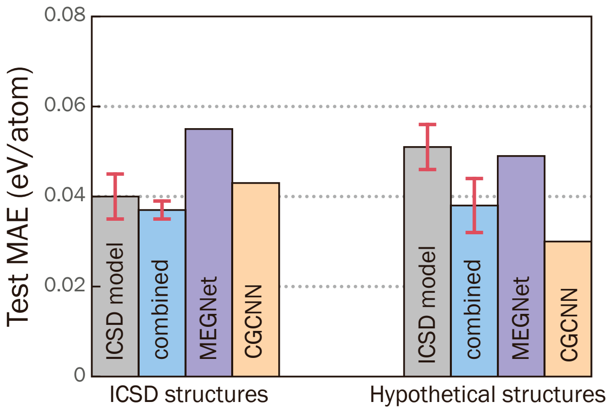

Figure S5: **Comparison of prediction accuracy:** Comparison of prediction MAE for ICSD and hypothetical structures of the model trained only on ICSD structures (Figure 1a in main text), model trained only on hypothetical structures (Figure 3a in main text), model trained on the combined dataset (blue), MEGNet model trained on the combined dataset (purple), and CGCNN model trained on the combined dataset (orange). For an equitable comparison, in all cases the same crystal structures are used in the training, validation, and test sets. The standard deviation (shown as error bars) is calculated from 4 different models with non-overlapping test sets.

7. Predicted Energy Rankings of MgO and ZnO Polymorphs

Figure 4 in the manuscript presents the energy rankings for different compositions in the hypothetical structures dataset. Here, we examine the energy rankings of two well-known binary compounds, MgO and ZnO, for which several experimentally realized and computationally proposed polymorphs are documented in the ICSD. There are 9 and 5 unique polymorphic structures reported for MgO and ZnO, respectively. The hybrid model correctly identifies the ground-state structures (rocksalt MgO, wurtzite ZnO) and also, satisfactorily ranks the other polymorphic structures.

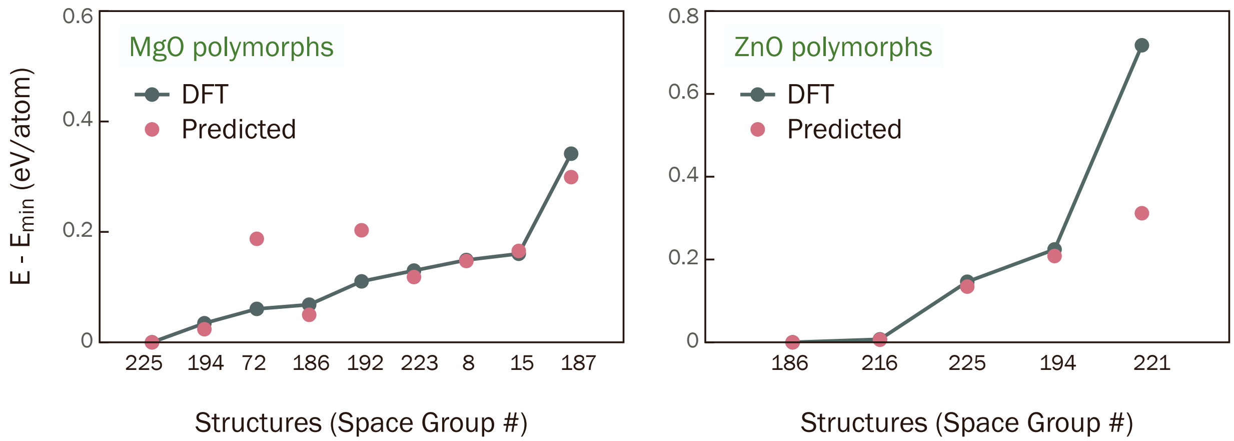

Figure S6: **Energy ranking of polymorphic structures:** Predicted relative energy $(E - E_{\mathrm{min}})$ of MgO and ZnO polymorphs reported in the ICSD is compared with DFT values. The model correctly identifies the known ground-state structures of both MgO (rocksalt, space group #225) and ZnO (wurtzite, space group #186).

8. Predicted Energy Ranking with GNN Models Trained on ICSD Structures

We train our GNN model, and MEGNet and CGCNN models on identical dataset consisting of only ICSD structures and compare the predicted energy rankings with DFT. The models consistently fail to rank structures in the correct order of their energies because of their bias towards the ground state structures. In particular, the models incorrectly label higher-energy structures as low energy.

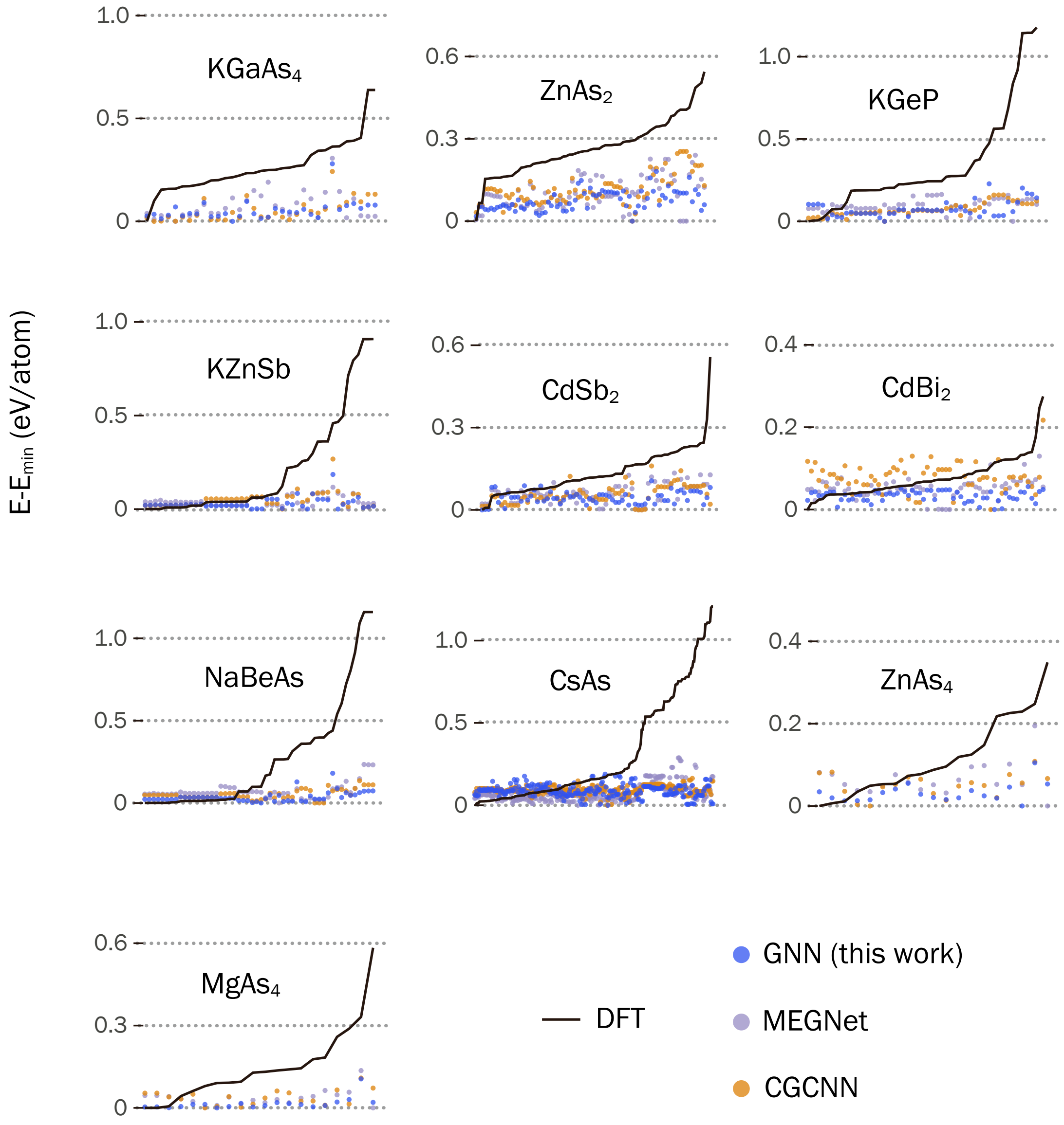

Figure S7: **Energy ranking of hypothetical structures:** Predicted relative energy ($E - E_{\text{min}}$) of hypothetical structures of 10 different compositions from the test set in Figure 3(b) compared with DFT, using the models trained on ICSD structures. The x-axes represent different polymorphic structures of a given composition, which are generated through ionic substitution.

9. k-Nearest Neighbor Analysis of Ca_7Ge

The total energy of Ca_7Ge is severely underestimated (-0.545 eV/atom relative to the DFT value) by the hybrid model (Figure 3b). The intermetallic compound Ca_7Ge lies above the convex hull (see manuscript for details). To understand the source of the error, we perform a k-Nearest Neighbor (kNN) analysis of the elemental embeddings for all Ca and Ge sites in Ca_7Ge. From this analysis, we identify the first 10 NNs and their elemental identities. The Ca(*4b*) Wyckoff site has 9 NNs that are Ba atoms, while 1 NN is Sr. In contrast, the Ca(*24d*) Wyckoff site has 3 Ca NNs, 2 Sr, and 5 Ba. Moreover, the Ca-Ge bond lengths associated with the Ca(*4b*) site are larger compared to the Ca(*24d*) site. The Ge(*4a*) site has all 10 NNs that are Ge.

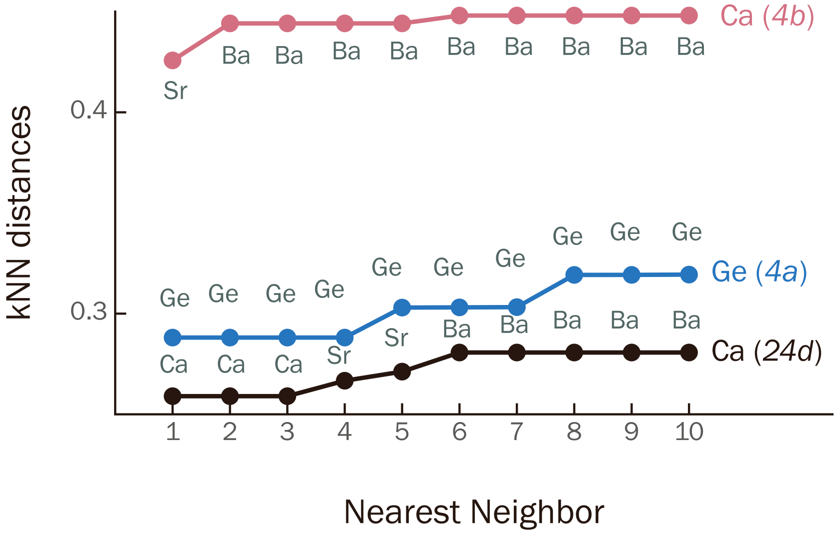

Figure S8: **Elemental nearest neighbor analysis of Ca_7Ge:** k-Nearest Neigbor (kNN) distances of the first 10 nearest neighbors for each Ca (*4b*, *24d* and Ge (*4a*) Wyckoff sites in Ca_7Ge. The elemental identities of the 10 nearest neighbors for each Wyckoff site are labelled.

10. Site Energies and t-SNE Projections: $Na_{17}Al_5O_{16}$, $Na_{14}Al_4O_{13}$

Chemical trends are identified by analyzing the probability density distribution of the elemental site energies (Figure 5) and t-SNE analyis of the elemental embeddings (Figure 6). In some cases, there can be a departure from the general trends. For example, some of the Na sites in $Na_{17}Al_5O_{16}$ (space group #8) and $Na_{14}Al_4O_{13}$ (space group #14) are 3-fold and 4-fold coordinated with elemental site energies in the "tail" of the oxides (near the peak of pnictides) energy distribution. The elemental embeddings for those same Na sites lie in the pnictogen cluster in the t-SNE projection. The other Na sites that lie closer to the peak of the oxides energy distribution (in the oxides cluster in t-SNE projection) are 5-fold coordinated.

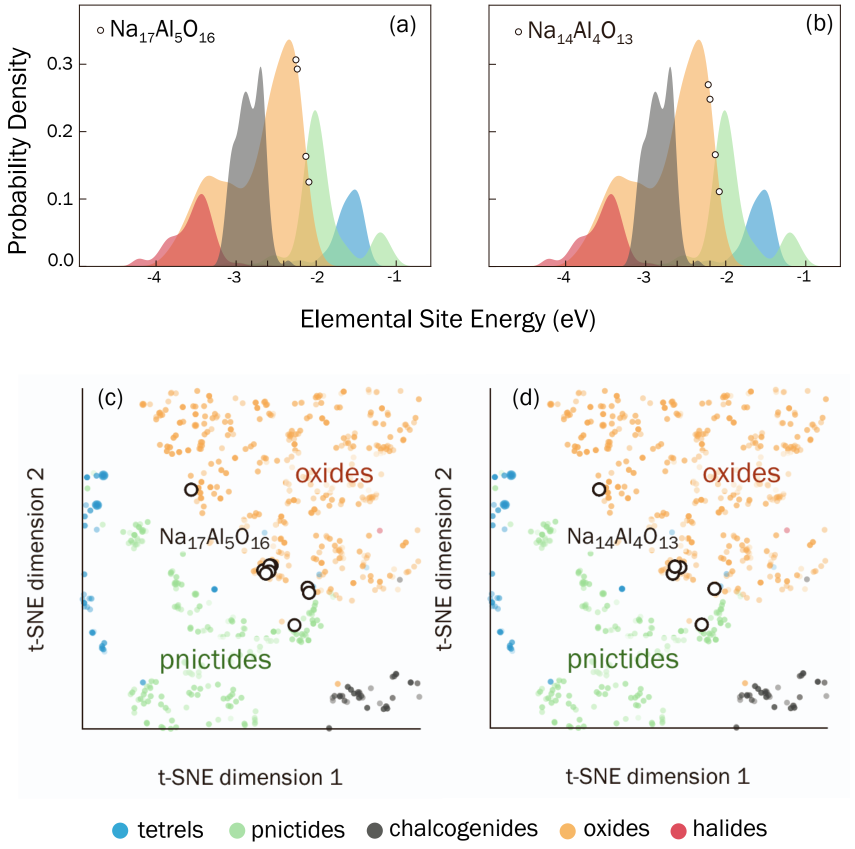

Figure S9: **Elemental site energies of $Na_{17}Al_5O_{16}$ and $Na_{14}Al_4O_{13}$:** Elemental site energy and t-SNE projection of elemental embedding of Na sites in (a, c) $Na_{17}Al_5O_{16}$ and (b, d) $Na_{14}Al_4O_{13}$ are marked with open circles.

11. Phase Stability Assessment with GNN Model

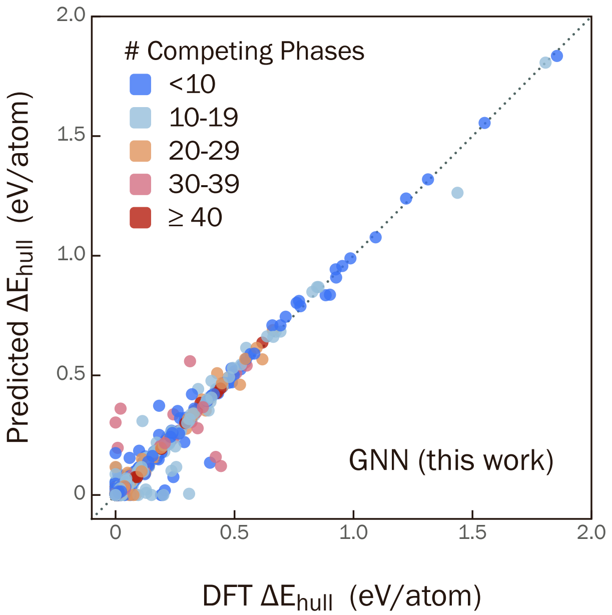

Figure S10: **Phase stability predicted with GNN:** Comparison of energy above the convex hull (ΔE_{hull}) predicted with our GNN model (trained on the combined dataset) and with DFT for 1794 ICSD compounds. The color scheme corresponds to the number of competing phases for each compound.

12. Phase Stability of Compounds Containing Group 5-10 Transition Metals

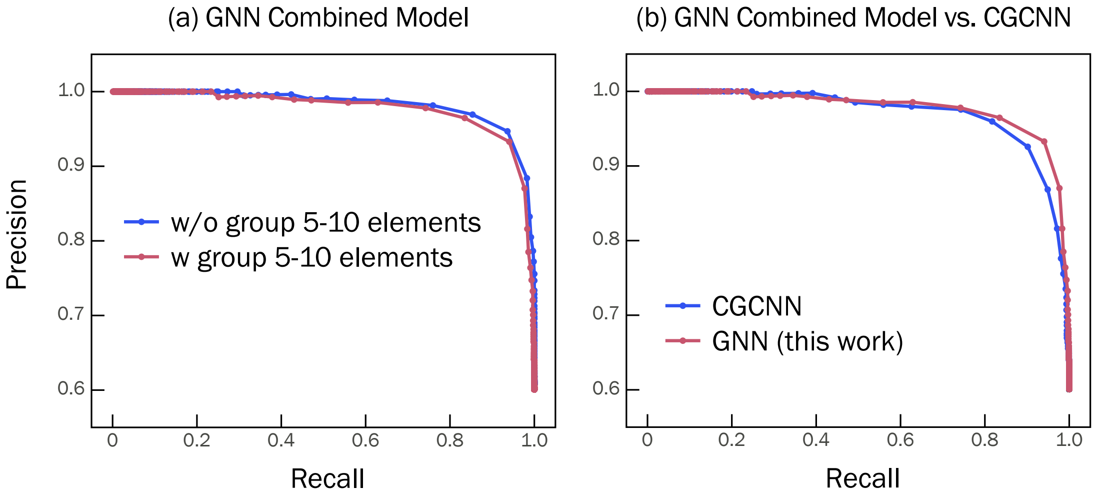

Figure S11: **Phase stability prediction of compounds with and without redox-active transition elements:** (a) PRC for phase stability predictions with our combined model compared for 761 compounds containing redox-active transition elements (groups 5-10) and 1033 compounds not containing groups 5-10 elements. (d) PRC for phase stability predictions of 1794 ICSD compounds (Figure S10) compared for our GNN model (trained on the combined dataset) and the CGCNN model (also, trained on the combined dataset). The performance of the re-trained CGCNN model is similar to our GNN model.

13. Comparison of ICSD and Combined Models in Predicting Phase Stability of Hypothetical Structures

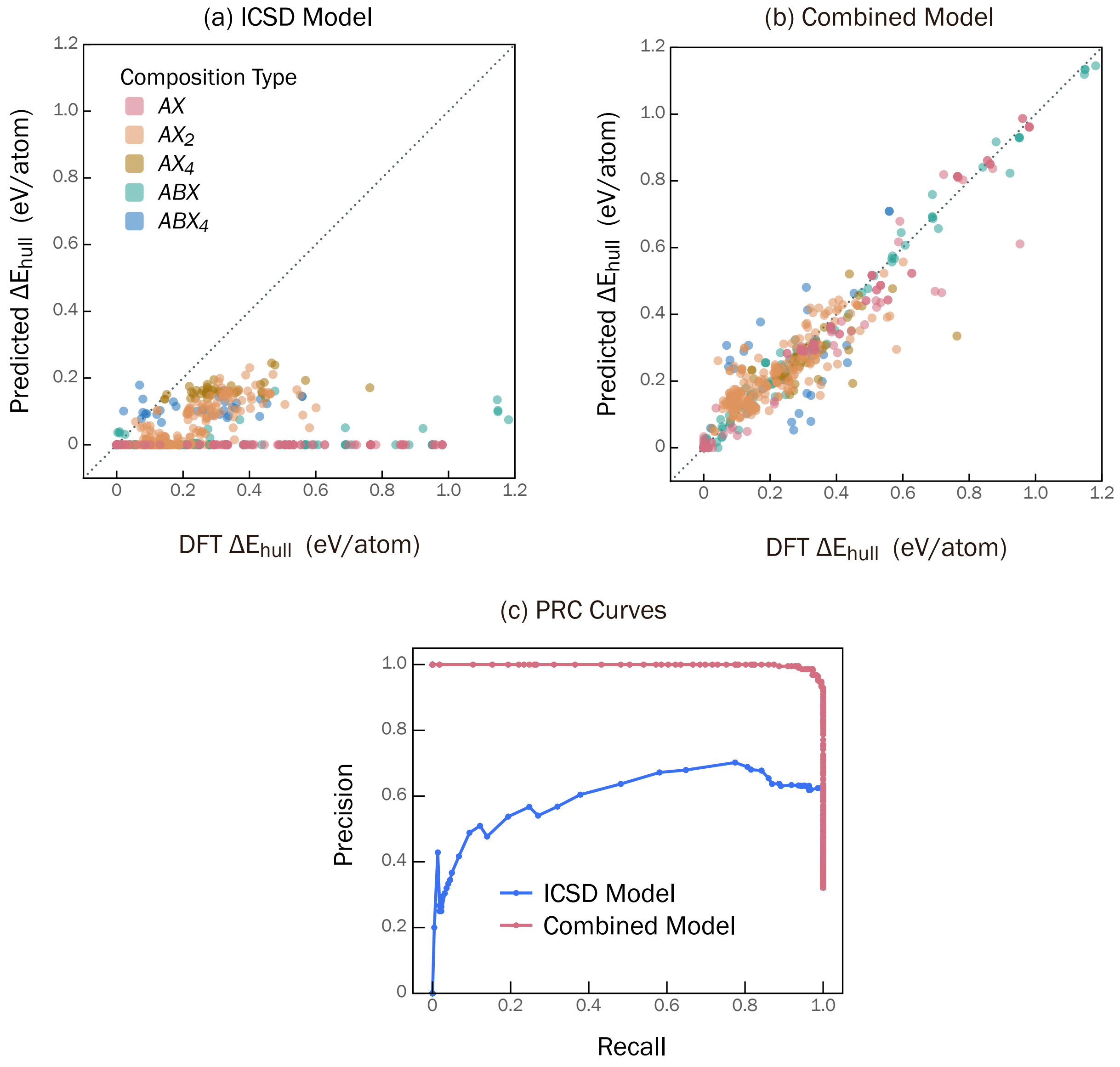

Figure S12: **Comparison of ICSD and combined models in predicting phase stability:** Phase stability of the hypothetical structures in Figure 4 of the main text. Predicted energy above the convex hull (ΔE_{hull}) compared with the corresponding DFT values for the (a) ICSD model, and (b) combined model. (c) Precision-Recall curves for the ICSD model and the combined model. Since the ICSD model is biased towards ground-state structures, it predicts significantly more false positives than the combined model. The area under the PRC curves (AU-PRC) for the ICSD and combined models are 0.60 and 0.99, respectively.

14. Comparison of GNN and Composition-only Models for Phase Stability

A precision-recall curve (PRC) provides a quantitative measure of the model's accuracy to classify a material as stable or unstable. The PRC for our GNN model prediction of phase stability of the 1033 compounds is compared with the PRC for stability predictions with the composition-based Magpie model. The area under the PRC (AU-PRC) is 1 for perfect classification and 0 for random guess. The AU-PRC for our GNN model is 0.98, which is significantly higher than that for Magpie model (0.78).

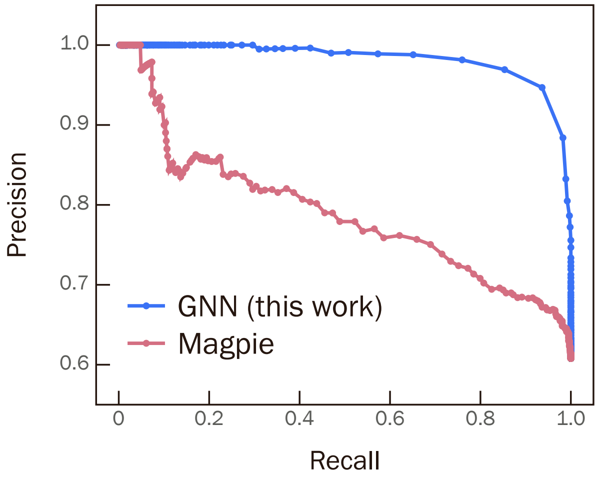

Figure S13: **Comparison of phase stability prediction with GNN and composition-only models:** Comparison of precision-recall curves (PRC) for phase stability predictions of 1033 ICSD compounds with our GNN model (trained on combined dataset) and the composition-based Magpie model.
